# ISL1 overexpression enhances the survival of transplanted human mesenchymal stem cells in a murine myocardial infarction model

**DOI:** 10.1186/s13287-018-0803-7

**Published:** 2018-02-26

**Authors:** Qiuling Xiang, Yan Liao, Hua Chao, Weijun Huang, Jia Liu, Haixuan Chen, Dongxi Hong, Zhengwei Zou, Andy Peng Xiang, Weiqiang Li

**Affiliations:** 1 Center for Stem Cell Biology and Tissue Engineering, Key Laboratory for Stem Cells and Tissue Engineering, Ministry of Education, Sun Yat-sen University, Guangzhou, People’s Republic of China; 2 Zhongshan Medical School, Sun Yat-sen University, Guangzhou, People’s Republic of China; 3 Department of Cardiology, the Red Cross hospital of Guangzhou City, the Fourth Affiliated Hospital of Jinan University, Guangzhou, People’s Republic of China; 4 The First Affiliated Hospital, Sun Yat-sen University, Guangzhou, People’s Republic of China; 5 Sun Yat-sen Memorial Hospital, Sun Yat-sen University, Guangzhou, People’s Republic of China

**Keywords:** ISL1, Human mesenchymal stem cells, Survival, Myocardial infarction

## Abstract

**Background:**

The LIM-homeobox transcription factor islet-1 (ISL1) has been proposed as a marker for cardiovascular progenitor cells. This study investigated whether forced expression of ISL1 in human mesenchymal stem cells (hMSCs) improves myocardial infarction (MI) treatment outcomes.

**Methods:**

The lentiviral vector containing the human elongation factor 1α promoter, which drives the expression of ISL1 (EF1α-ISL1), was constructed using the Multisite Gateway System and used to transduce hMSCs. Flow cytometry, immunofluorescence, Western blotting, TUNEL assay, and RNA sequencing were performed to evaluate the function of ISL1-overexpressing hMSCs (ISL1-hMSCs).

**Results:**

The in vivo results showed that transplantation of ISL1-hMSCs improved cardiac function in a rat model of MI. Left ventricle ejection fraction and fractional shortening were greater in post-MI hearts after 4 weeks of treatment with ISL1-hMSCs compared with control hMSCs or phosphate-buffered saline. We also found that ISL1 overexpression increased angiogenesis and decreased apoptosis and inflammation. The greater potential of ISL1-hMSCs may be attributable to an increased number of surviving cells after transplantation. Conditioned medium from ISL1-hMSCs decreased the apoptotic effect of H_2_O_2_ on the cardiomyocyte cell line H9c2. To clarify the molecular basis of this finding, we employed RNA sequencing to compare the apoptotic-related gene expression profiles of control hMSCs and ISL1-hMSCs. The results showed that insulin-like growth factor binding protein 3 (IGFBP3) was the only gene in ISL1-hMSCs with a RPKM value higher than 100 and that the difference fold-change between ISL1-hMSCs and control hMSCs was greater than 3, suggesting that IGFBP3 might play an important role in the anti-apoptosis effect of ISL1-hMSCs through paracrine effects. Furthermore, the expression of IGFBP3 in the conditioned medium from ISL1-hMSCs was almost fourfold greater than that in conditioned medium from control hMSCs. Moreover, the IGFBP3 neutralization antibody reversed the apoptotic effect of ISL1-hMSCs-CM.

**Conclusions:**

These results suggest that overexpression of ISL1 in hMSCs promotes cell survival in a model of MI and enhances their paracrine function to protect cardiomyocytes, which may be mediated through IGFBP3. ISL1 overexpression in hMSCs may represent a novel strategy for enhancing the effectiveness of stem cell therapy after MI.

**Electronic supplementary material:**

The online version of this article (10.1186/s13287-018-0803-7) contains supplementary material, which is available to authorized users.

## Background

Heart disease is a major cause of mortality and morbidity worldwide. Acute and chronic loss of cardiomyocytes is a main contributor to poor cardiac function that culminates in heart failure [1]. Investigators have long pursued strategies to replace lost heart muscle. One approach has been to isolate stem cells from bone marrow, blood, skeletal muscle, or fat and inject them into the damaged heart [2]. This strategy has been tested for safety and efficacy in animal models of myocardial infarction (MI) and humans. However, one of the major obstacles to using stem cells for therapy is the poor survival rate of transplanted stem cells in ischemic tissues due to the hypoxic environment [3]. Interestingly, Gnecchi et al. provided evidence that a combination of human mesenchymal stem cells (hMSCs) and AKT overexpression-based gene therapy enhanced cell survival, therapeutic neovascularization, myocardial protection, and functional improvement through paracrine mechanisms [4]. Another report showed that prolyl hydroxylase domain protein 2 (PHD2) silencing enhanced the survival and paracrine function of transplanted adipose-derived stem cells in infarcted myocardium [5].

The LIM-homeobox transcription factor islet-1 (ISL1) plays a crucial role during heart embryogenesis and later promotes the development of adult resident cardiac stem cells. One of the earliest steps in cardiogenesis is when the cells of the cardiogenic mesoderm form the cardiac crescent. ISL1 progenitors, which comprise the cardiac crescent, migrate into the developing heart and give rise to the outflow tract, right ventricle, and both atria [6, 7]. ISL1 expression is then turned off except in a small population of resident cardiac stem cells which maintain ISL1 expression throughout adulthood [8]. Cagavi et al. described functional cardiomyocytes derived from ISL1 cardiac progenitors via bone morphogenetic protein stimulation, which could lead to enhancement of cardiac differentiation and engraftment [9]. Our previous work confirmed that ISL1 overexpression in hMSCs promotes vascularization through monocyte chemoattractant protein-3 [10]. However, whether ISL1 overexpression influences the effects of hMSC transplantation in infarcted myocardium remains unknown.

The purpose of this study was to test the hypothesis that ISL1 overexpression enhances stem cell survival and paracrine function after transplantation, thus enhancing protection and repair of the damaged heart. We constructed a lentivector for ISL1 overexpression under the control of the human elongation factor 1α (EF1α) promoter. This construct was used to generate ISL1-hMSCs, which were then injected into an MI model. The experiments performed showed that ISL1-hMSC administration improved cardiac function, and ISL1 overexpression enhanced cardioprotection by promoting hMSC survival in MI and enhancing their paracrine function, which may be mediated through insulin-like growth factor binding protein 3 (IGFBP3).

## Methods

### Cell culture

hMSCs were obtained from Cyagen Biosciences, Inc. (Guangzhou, China), and cultured in 90% Dulbecco’s modified Eagle medium (DMEM; Hyclone, Logan, UT, USA) supplemented with 10% fetal bovine serum (FBS; Invitrogen, Carlsbad, CA, USA), 100 IU/ml penicillin (Hyclone, Logan, UT, USA), and 100 μg/ml streptomycin (Hyclone, Logan, UT, USA). The medium was changed every 2 days. After reaching 90% confluence, cells were detached by incubating with 0.125% trypsin for 1–2 min at 37 °C and replated for continuous passage. Cells between 5 and 10 passages were used for all experiments. The rat cardiomyocyte cell line H9c2 was purchased from the Laboratory Animal Center of Sun Yat-sen University. The human cardiomyocyte cell line AC16 was kindly supplied by Drs. Yangxin Chen and Yong Xie, Sun Yat-sen Memorial Hospital [11]. Cells were maintained in DMEM supplied with 10% FBS and 1% antibiotic solution.

### Construction of ISL1-hMSCs

The detail of construction and characterization of ISL1-hMSCs are described in our prior publication [10]. In brief, entry vectors were generated by flanking the human EF1α promoter and human ISL1 gene with attB4/B1r and attB1/B2 sites, respectively, by polymerase chain reaction (PCR). The promoter PCR product was cloned into pDONR P4-P1r (Invitrogen, Carlsbad, CA, USA) using the Gateway BP recombination method. The att-flanked ISL1 fragment was cloned into pDONR 221 (Invitrogen, Carlsbad, CA, USA) using the same method. The resulting vectors, termed pUp-EF1α and pDown-ISL1, were recombined into the pDest-puro vector using a recognized LR recombination reaction protocol described in the Gateway LR kit and a clonase enzyme mix. The final lentiviral expression vector was designated pLV/puro-EF1α-ISL1 (EF1α-ISL1). The control vector, pLV/puro-EF1α-empty (EF1α-Empty) was transduced into hMSCs as a control.

Lentiviruses were prepared by transient cotransfection of 293FT cells with the EF1α-ISL1 construct or EF1α-Empty together with the ViraPower Lentiviral packaging mix (Invitrogen, Carlsbad, CA, USA) using Lipofectamine 2000 (Invitrogen, Carlsbad, CA, USA). Three days after transfection, supernatants containing viral particles were harvested, filtered through polyether sulfone membranes (pore size 0.45 μm), and titered.

For lentiviral transduction, hMSCs were dissociated into single-cell suspensions using 0.125% TrypLE Select (Invitrogen, Carlsbad, CA, USA) and then replated with lentiviral particles and 5 μg/ml polybrene (Sigma-Aldrich, St. Louis, MO, USA). The medium was replaced with fresh culture medium 12–24 h after infection. Four days after transduction, puromycin (Sigma-Aldrich, St. Louis, MO, USA) was added to the culture medium at a concentration of 1–5 μg/ml, and the cells were maintained in this medium for 5 days. Ultimately, ISL1-hMSCs (ISL1 overexpression hMSCs) and Ctrl-hMSCs (empty vector control hMSCs) were obtained.

### Characterization of ISL1-hMSCs

ISL1-hMSCs or Ctrl-hMSCs were analyzed by flow cytometry with primary fluorescence using fluorescein isothiocyanate (FITC)-conjugated (anti-CD29, anti-CD34, and anti-CD45) or phycoerythrin (PE)-conjugated (anti-CD14, anti-CD31, anti-CD73, and anti-CD90) antibodies (BD Biosciences, San Diego, CA). Irrelevant isotype-matched antibodies (BD Biosciences, San Diego, CA) were used as negative controls. Flow cytometry analyses were performed with an Influ cell sorter (BD Biosciences, San Diego, CA). The data were analyzed using FlowJo7.5 software (Treestar, Ashland, OR, USA).

### Stem cell differentiation assay

For osteogenic differentiation, the cells were incubated in osteogenic medium containing 10 μM dexamethasone, 0.2 mM ascorbic acid, and 10 mM β-glycerophosphate (Sigma-Aldrich, St. Louis, MO, USA). The medium was replaced twice weekly. After 14 days in differentiation medium, the cells displayed bone-like nodular aggregates of matrix mineralization. Mineral deposition was visualized by Alizarin Red S staining for calcium.

For chondrogenic differentiation, cells were cultured in complete medium supplemented with 100 μg/mL sodium pyruvate, 10 ng/mL transforming growth factor (TGF)-β1, 100 nM dexamethasone, 1% insulin-transferrin-selenium, and 100 μg/mL ascorbate-2-phosphate. To evaluate the deposition of glycosaminoglycans, after 14 days the cells were fixed with 10% formalin for 20 min and stained with Alcian Blue 8GX (Cyagen Biosciences, Guangzhou, China) for 30 min.

For adipogenesis [12], induction and maintenance media were used alternately for 3 days. The induction medium consisted of DMEM (high glucose), 10% FBS, 1% antibiotic solution, 0.5 mM 1-methyl-3-isobutylxanthine (IBMX), 200 mM indomethacin, 10 mg/ml insulin, and 0.1 mM dexamethasone (Sigma-Aldrich, St. Louis, MO, USA). The maintenance medium consisted of DMEM, 10% FBS, 1% antibiotic solution, and 10 mg/ml insulin. Adipogenic differentiation was analyzed by Oil-Red O staining (Sigma-Aldrich, St. Louis, MO, USA) after induction for 2–3 weeks.

### Quantitative reverse-transcription PCR (qRT-PCR)

Total RNA was extracted from cells using TRIzol Reagent (Invitrogen, Carlsbad, CA, USA). Samples (1 μg) of total RNA were reverse-transcribed using a First Strand complementary DNA synthesis kit for RT-PCR (Roche, Indianapolis, IN, USA). qRT-PCR was performed using SYBR PCR Master Mix (Toyobo, Japan) according to the manufacturer’s instructions. qRT-PCR was conducted in duplicate for each sample, and three independent experiments were performed. Signals were detected using a Light Cycler 480 detection system (Roche, Indianapolis, IN, USA). The primer sequences are listed in Additional file 1: Table S1.

### Myocardial infarction studies

Three-month-old Sprague-Dawley rats were used to establish an MI model, as previously described [13]. The Sprague-Dawley rats were supplied by the Animal Center, Sun Yat-sen University. The animal study was approved by the Animal Care and Use Committee of Sun Yat-sen University. Rats (37 in total, five rats died during or after the surgery) were divided into four groups: the sham group (*n* = 8), the control group (*n* = 8), the Ctrl-hMSCs group (*n* = 8), and the ISL1-hMSCs group (*n* = 8). Briefly, the rats were anesthetized with ketamine (100 mg/kg intraperitoneally) prior to undergoing a left intercostal thoracotomy. After the left anterior descending coronary artery (LAD) was identified it was ligated directly below the left atrial appendage with 8-0 nylon sutures. Abnormalities in the pallor and regional wall motion of the left ventricle confirmed the occlusion. In some groups, a total of 10^6^ CM-Dil-labeled cells (in 50 μL DMEM) or 50 μL DMEM alone was injected intramuscularly into two sites of the ischemic border zone. The chest wall was then closed, the lungs were inflated, the rat was extubated, and the tracheotomy was closed. After recovery, the rats were returned to the animal facility for 1–28 days. The ligated hearts were harvested at different time intervals after LAD ligation (7 and 28 days) and embedded in optimal cutting temperature (OCT) compound (Sakura Finetek, Torrance, USA). Frozen sections (10 μm) were collected for each whole heart and prepared for immunofluorescence staining. For histological staining, the ligated hearts were fixed in 4% paraformaldehyde and embedded in paraffin. The sections were stained using Masson’s trichrome stain.

### Echocardiography analysis

Echocardiography was performed (GE vivid 7 dimension) to determine cardiac structure and function in conscious rats. Hearts were viewed in the short-axis between the two papillary muscles, and each measurement was obtained in M-mode by averaging the results from three consecutive heart beats. Fractional shortening (FS) and the left ventricular ejection fraction (EF) were automatically calculated using the echocardiography software according to the Teicholz formula. Parameters were measured to determine structural changes in cardiac morphology. The individuals performing the echocardiography were blinded to the animal treatments.

### Immunofluorescence and immunohistochemistry

Prior to immunofluorescence analyses, cells were fixed in 4% (v/v) paraformaldehyde for 20 min and then permeabilized by incubation for 30 min at room temperature in phosphate-buffered saline (PBS) containing 0.1% (v/v) Triton X-100, goat serum, and 1% (w*/*v) bovine serum albumin (BSA; Sigma). Cells were incubated overnight at 4 °C with primary antibodies against ISL1 (1:300, cat. no. ab178400, Abcam, Cambridge, UK), α-actinin (1:200, cat. no. ab50599, Abcam, Cambridge, UK) to detect cardiomyocytes, CD3 (1:300, cat. no. ab16669, Abcam, Cambridge, UK) to detect T lymphocytes, and CD68 (1:300, cat. no. ab125212, Abcam, Cambridge, UK) to detect macrophages. Inflammation factors interleukin (IL)-6 (1:400, cat. no. Ab9324, Abcam, Cambridge, UK), IL-10 (1:100, cat. no. Ab9969, Abcam, Cambridge, UK), and tumor necrosis factor (TNF)-α (1:100, cat. no. Ab6671, Abcam, Cambridge, UK) were also detected. Rabbit IgG secondary antibody, Alexa Fluor 594 (1:400, cat. no. A-21207, Invitrogen, Carlsbad, CA, USA) or mouse IgG secondary antibody, Alexa Fluor 594 (1:400, cat. no. A-21203, Invitrogen, Carlsbad, CA, USA) were added, and the incubation was performed at room temperature for 1 h in the dark. Nuclei were counterstained with 4’6-diamidino-2-phenylindole (DAPI; 1:1000, cat. no. D9542, Sigma-Aldrich, St. Louis, MO, USA).

Formalin-fixed and paraffin-embedded samples were deparaffinized and rehydrated. After rinsing with PBS, antigen retrieval was carried out by microwave treatment in 0.01 M sodium citrate buffer (pH 6.0) at 100 °C for 15 min. Antibody incubations were as follows: CD31 antibody (1:400, cat. no. Ab119339, Abcam, Cambridge, UK) was used to detect capillaries; human anti-nuclei antibody (HNA; 1:400, cat. no. MAB1281, Millipore, Bedford, MA, USA) was used to track grafted cells. For immunohistochemistry assessment, nuclei counterstaining was performed and brown-yellow staining was considered positive expression.

### Cell survival assay

The influence of ISL1 transduction on hMSC survival was determined by seeding 1 × 10^5^ ISL1-hMSCs or control cells per well in a six-well plate. After incubation overnight, media were replaced with serum-free DMEM, and the cells were cultured for an additional 48 h. ISL1-hMSCs and Ctrl-hMSCs were treated with 100 μM H_2_O_2_ for 8 h. Cell survival was determined by fluorescence-activated cell sorting (FACS) using Annexin V/propidium iodide (PI) antibodies. The data were analyzed using FlowJo7.5 software (Treestar, Ashland, OR, USA).

### Conditioned medium (CM) preparation

CM was prepared according to our previous reports [14]. After the Ctrl-hMSCs or ISL1-hMSCs had reached greater than 80% confluence, the cells were cultured with serum-free DMEM for another 24 h. The medium from 10^6^ cells yielded 6 ml of primary CM, which was further de-salted and concentrated 30-fold by centrifugation (4000 × g for 30 min at 4 °C) using ultrafiltration units with a 3-kDa molecular weight cutoff (Amicon Ultra-PL 3; Millipore, Billerica, MA, USA), yielding 200 μl of concentrated CM. Serum-free DMEM (de-salted and concentrated 30-fold) served as a vehicle control. CM was used immediately or stored at −80 °C.

### IGFBP3 inhibition assay

For the neutralization of IGFBP3 in CM, different concentrations of anti-IGFBP3 antibody (IGFBP3-Ab, R&D Systems, Minneapolis, MN, USA) were added: 0.25 μg/mL, 0.5 μg/mL, 1 μg/mL, 1.5 μg/mL, and 2 μg/mL IGFBP3-Ab was preincubated in medium for 30 min. Then, CM was added. Cells with or without Ctrl-hMSCs-CM or ISL1-hMSCs-CM were subjected to 200 μM H_2_O_2_ for 6 h to detect apoptosis.

### Transferase-mediated deoxyuridine triphosphate-biotin nick end labeling (TUNEL assay)

To detect cardiomyocyte apoptosis, an in-situ apoptotic cell death detection kit (cat. no. 11684795910, Roche, Indianapolis, IN, USA) based on the TUNEL assay was used. For the in vivo experiment, paraffin sections of heart were stained with TUNEL reagent. Brown-yellow staining in the nuclei was considered positive expression for immunohistochemistry assessment. Green fluorescence was positive for the immunofluorescence assay.

For the in vitro experiment, the cardiomyocyte cell line H9c2 with or without Ctrl-hMSCs-CM or ISL1-hMSCs-CM was subjected to 200 μM H_2_O_2_ for 6 h. Cell nuclei were stained with DAPI (blue) and TUNEL positive (green). The percentage of apoptotic nuclei per section was calculated by counting the number of TUNEL staining cardiomyocyte nuclei divided by the total number of DAPI-positive nuclei.

### Western blotting

Cell lysates with equal total protein amounts were separated by SDS-PAGE gel. Proteins were transferred electrophoretically to polyvinylidene difluoride (PVDF) membranes (Bio-Rad). The membranes were blocked in 5% milk in PBS-T (0.1% Tween20) at room temperature for 1 h. The membranes were probed with primary antibody specific for caspase 3, cleaved caspase 3, caspase 9, cleaved caspase 9, Bax, Bcl2 (1:1000, Cell Signaling Technology), or β-actin (1:10,000, Cell Signaling Technology). The primary antibody was then identified by a horseradish peroxidase (HRP)-conjugated secondary antibody diluted 1:5000 (Cell Signaling Technology). Finally, the membranes were developed using an enhanced chemiluminescence (ECL) advance detection kit (GE healthcare) and exposed to x-ray films. The band density was analyzed using ImageJ software (Rawak Software, Inc., Germany).

### RNA sequencing

Ctrl-hMSCs or ISL1-hMSCs were prepared. RNA library preparation and sequencing were performed as recommended by the manufacturer (Genome Analyzer IIx, Illumina, USA). Sequencing data were processed using Consensus Assessment of Sequence and Variation software (Illumina) with default settings. Genes with RPKM (reads per kilobase of exon per million mapped reads) values of more than five were enrolled in the functional analysis according to the Gene Ontology (GO) database. Genes related to apoptosis were selected to draw a heatmap with HemI (Heatmap Illustrator, Huazhong University of Science and Technology, China).

### Enzyme-linked immunosorbent assay (ELISA)

The levels of IGFBP3 in the conditioned medium of Ctrl-hMSCs or ISL1-hMSCs were determined by ELISA using commercially available Quantikine kits (R&D Systems, Minneapolis, MN, USA) according to the manufacturer’s instructions. All samples and standards were measured in duplicate.

### Proliferation assay

Isolated CD3^+^ T lymphocytes were stained using a CellTrace CFSE cell proliferation kit (5 mmol/L; Invitrogen, Carlsbad, CA, USA) according to the manufacturer’s instructions. Labeled CD3^+^ T cells were cultured in replicate wells with or without hMSCs and stimulated with an anti-CD3 monoclonal antibody (0.2 mg/mL, BD Biosciences, San Diego, CA) and an anti-CD28 monoclonal antibody (1 mg/mL, BD Biosciences, San Diego, CA) for 96 h. T-cell proliferation was evaluated by flow cytometric analysis of 5,6-carboxyfluorescein diacetate succinimidyl ester (CFSE) dilutions.

### Intracellular cytokine staining

CD3^+^ T cells were sorted from healthy donors, resuspended in RPMI 1640 medium (Hyclone, Logan, UT, USA), and cultured in duplicate wells with or without Ctrl-hMSCs or ISL-hMSCs for 72 h. Brefeldin A (BFA; 10 mg/mL, Sigma-Aldrich, St. Louis, MO, USA), phorbol-12-myristate-13-acetate (PMA; 50 ng/mL, Sigma-Aldrich, St. Louis, MO, USA), and ionomycin (1 mg/mL, Sigma-Aldrich, St. Louis, MO, USA) were added, and the cells were cultured for an additional 6 h. The cells were fixed, permeabilized, and cell-surface stained using v450-conjugated CD3 (CD3-v450), PE-conjugated TNFα-PE, and PE-cyanine 7-conjugated interferon-γ (IFN-γ-PE-Cy7). All three antibodies were purchased from BD Biosciences (San Diego, CA, USA).

### Statistical analysis

All results represent data collected from at least three independent experiments. Statistical analyses were performed using one-way analyses of variance (ANOVA). Post-hoc tests were used for statistical tests. A *p* value < 0.05 was considered a statistically significant difference. All data are expressed as the means ± standard deviation (SD).

## Results

### Expression of ISL1 in hMSCs

**Fig. 1:**
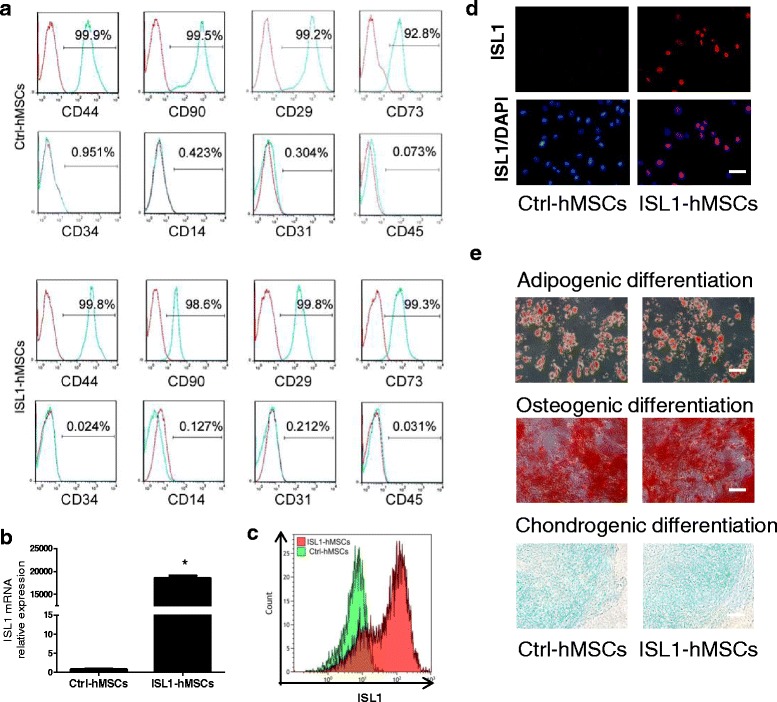
Characterization of islet-1 human mesenchymal stem cells (ISL1-hMSCs). Flow cytometric analysis (**a**) showed that ISL1-hMSCs were positive for CD29, CD44, CD73, and CD90, and negative for CD14, CD31, CD34, and CD45. qPCR (**b**) showed that lentiviral transduction of hMSCs with ISL1 specifically increased the expression of ISL1. Flow cytometry (**c**) and immunofluorescence staining (**d**) further confirmed the overexpression of ISL1. Adipogenic differentiation of ISL1-hMSCs was determined by histochemical staining of adipocytes (Oil-Red O), osteogenic differentiation of ISL1-hMSCs was determined by histochemical staining of osteocytes (Alizarin Red), and chondrogenic differentiation was detected by Alcian Blue (**e**). Data are presented as the mean ± SD, *n* = 3; *p < 0.05 vs. Ctrl-hMSCs. Scale bar = 50 μm. Ctrl control, DAPI 4’6-diamidino-2-phenylindole

The lentiviral vector EF1α-ISL1 was constructed and viruses were produced as described in the Methods. The ISL1-hMSCs were generated by transducing wild-type hMSCs with ISL1 by exposure to EF1α-ISL1 lentivirus for 24 h. After antibiotic selection, purified ISL1-hMSCs were obtained. The ISL1-hMSCs were characterized by multipotent differentiation potential and surface markers. Flow cytometry demonstrated that these cells were positive for the stem cell markers CD29, CD44, CD73, and CD90, but negative for the hematopoietic stem cell markers CD14, CD31, CD34, and CD45, indicating that transduced cells maintained the hMSC phenotype (Fig. 1a). Analysis by qRT-PCR showed that ISL1 overexpression was successfully achieved in hMSCs and that they had long-term stability in in-vitro cultures (Fig. 1b). Flow cytometry (Fig. 1c) and immunofluorescence staining (Fig. 1d) further confirmed the successful overexpression of ISL1 in hMSCs. The ISL1-hMSCs maintained the potential to differentiate into adipocytes, osteoblasts, and chondrocytes, demonstrating their multilineage differentiation potential. After specific induction, ISL1-hMSCs or Ctrl-hMSCs were positive for Alizarin Red, Oil-Red O and Alcian Blue staining (Fig. 1e).

### ISL1-hMSC transplantation improved cardiac function and attenuated myocardial infarct size

**Fig. 2:**
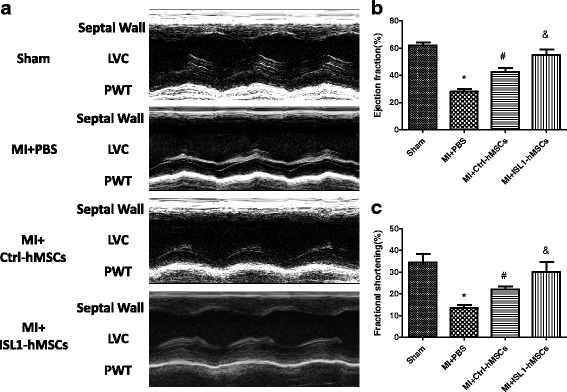
Transplantation of islet-1 human mesenchymal stem cells (ISL1-hMSCs) improved cardiac function in a murine myocardial infarction (MI) model. Representative M-mode images (**a**) of hearts with sham surgery or MI at 4 weeks after phosphate-buffered saline (PBS), control (Ctrl)-hMSC, or ISL1-hMSC injection. Ejection fraction (**b**) and fractional shorting (**c**) at 4 weeks after ISL1-hMSC or Ctrl-hMSC transplantation were detected. Data are presented as the mean ± SD, *n* = 8; *p < 0.05, vs. Sham; ^#^*p* < 0.05 vs. MI + PBS; ^&^*p* < 0.05 vs. MI + ISL1-hMSCs. LVC left ventricular cavity, PWT posterior wall thickness

**Fig. 3:**
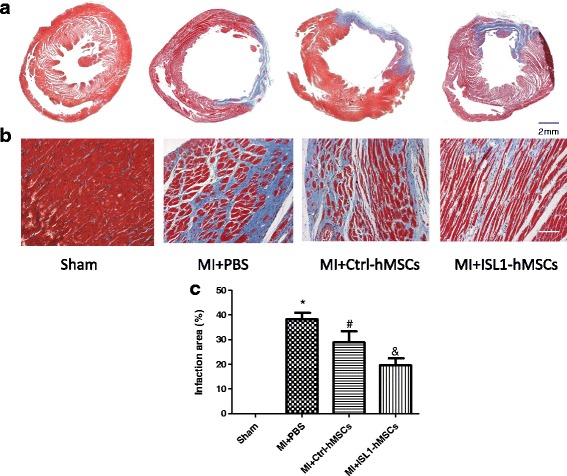
Transplantation of islet-1 human mesenchymal stem cells (ISL1-hMSCs) reduced cardiac infarct size and fibrotic size in a myocardial infarction (MI) model. (**a**) Representative images of infarct size. Representative images (**b**) and quantification (**c**) of the fibrotic area in the infarct border zone by Masson’s trichrome staining at 4 weeks post-MI. Data are presented as the mean ± SD, n = 8; *p < 0.05, vs. sham; ^#^*p* < 0.05 vs. MI + phosphate-buffered saline (PBS); ^&^*p* < 0.05 vs. MI + ISL1-hMSCs. Scale bar = 50 μm. Ctrl control

To determine whether ISL1 enhances the therapeutic effect of stem cells, we evaluated the effects of hMSC transplantation on cardiac function and infarct size in post-MI rat hearts. Left ventricle EF and FS were greater in post-MI hearts at 4 weeks treated with ISL1-hMSCs than in Ctrl-hMSCs or PBS (Fig. 2). EF in the ISL1-hMSC group increased to 55.19 ± 3.87% (*p* < 0.05 vs. MI at 28.03 ± 4.10%; *p* < 0.05 vs. Ctrl-hMSCs at 42.43 ± 3.03%). FS in the ISL1-hMSC group increased to 30.20 ± 4.41% (*p* < 0.05 vs. MI at 13.49 ± 3.10%; *p* < 0.05 vs. Ctrl-hMSCs at 22.01 ± 3.04%). Left ventricular end-diastolic volume (LVEDV) and left ventricular end-systolic volume (LVESV) were greater in post-MI hearts after 4 weeks of treatment with ISL1-hMSCs than PBS (Additional file 2: Figure S1a, b). Cardiac output (CO) in the sham group was 78.49 ± 6.07 ml/min, and it decreased to 46.41 ± 6.19 ml/min in the MI group. In the Ctrl-hMSC group, CO increased to 59.36 ± 6.54 ml/min (*p* < 0.05 vs MI). In the ISL1-hMSC group, it recovered to 71.23 ± 6.66 ml/min (*p* < 0.05 vs MI) (Additional file 2: Figure S1c). The myocardial infarct size was significantly smaller in rat hearts treated with ISL1-hMSCs than in those with Ctrl-hMSCs or PBS at 4 weeks post-MI (Fig. 3a). Masson’s trichrome staining showed an increase in the islands of viable cardiac muscle in the peri-infarct regions at 4 weeks after Ctrl-hMSC or ISL1-hMSC treatment (Fig. 3b). The percentage of the fibrotic area in the total peri-infarct zone was significantly reduced in hMSC-treated hearts, and ISL1-hMSCs further reduced the fibrotic area (Fig. 3c).

### ISL1 overexpression increased angiogenesis and decreased apoptosis and inflammation

**Fig. 4:**
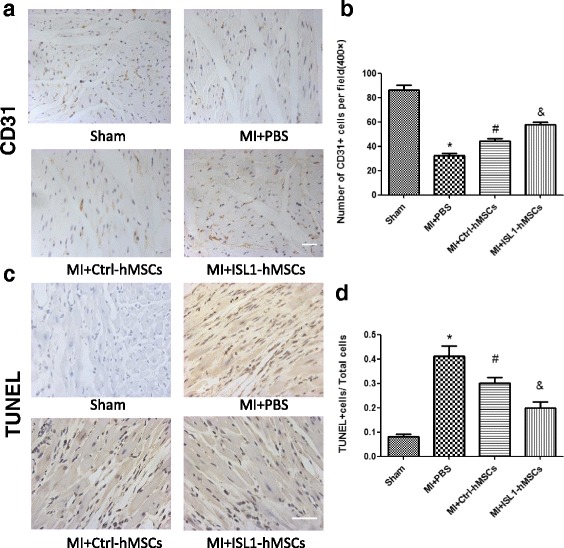
Transplantation of islet-1 human mesenchymal stem cells (ISL1-hMSCs) increased angiogenesis and reduced apoptosis. Representative images (**a**) and quantification (**b**) of capillary density in the infarct border zone at 4 weeks post-myocardial infarct (MI) detected by CD31 staining. Representative images (**c**) and quantification (**d**) of TUNEL-positive cells in the infarct border. Data are presented as the mean ± SD, n = 8; *p < 0.05, vs. sham; ^*#*^*p* < 0.05 vs. MI + phosphate-buffered saline (PBS); ^&^*p* < 0.05 vs. MI + ISL1-hMSCs. Scale bar = 50 μm. Ctrl control

We also investigated the effects of ISL1-hMSCs on angiogenesis and inflammation in post-MI hearts. At 4 weeks after transplantation, immunostaining showed more CD31-expressing capillaries present in peri-infarct regions of ISL1-hMSC- versus Ctrl-hMSC-treated hearts (Fig. 4a, b), which suggested that ISL1-hMSC transplantation promoted angiogenesis.

The TUNEL assay was used to measure apoptotic cell death in the infarct border zone. The TUNEL-positive nucleus number was moderately reduced in Ctrl-hMSC-treated hearts and more significantly reduced in ISL1-hMSC-treated hearts (Fig. 4c, d). We also used TUNEL/α-actinin double staining to detect positive apoptotic cardiomyocytes and found that ISL1-hMSC transplantation reduced TUNEL-positive cardiomyocytes compared to Ctrl-hMSCs or PBS (Additional file 2: Figure S2).

Inflammatory cell infiltration was analyzed by immunofluorescence after MI. ISL1-hMSCs reduced the amount of CD3-positive T cells (Additional file 2: Figure S3) and CD68-positive macrophages (Additional file 2: Figure S4). We also detected the inflammatory cytokines IL-6, IL-10, and TNFα in heart slides. ISL1-hMSC transplantation reduced the expression of inflammatory cytokines compared with the Ctrl-hMSC or PBS groups (Additional file 2: Figure S5). Furthermore, we analyzed the immunoregulatory effects of ISL1-hMSCs on the proliferation and proinflammatory cytokine production of CD3^+^ T cells in vitro. The results indicated that ISL1-overexpressing hMSCs markedly inhibited the proliferation of CD3^+^ T cells and decreased the percentages of TNFα-producing and IFN-γ producing CD3^+^ T cells, respectively, compared with the Ctrl-hMSC or PBS groups (Additional file 2: Figure S6), indicating that the enhanced therapeutic effect of ISL1-hMSCs might be partially associated with a reduced inflammatory response.

### ISL1 overexpression increased transplanted cell survival

**Fig. 5:**
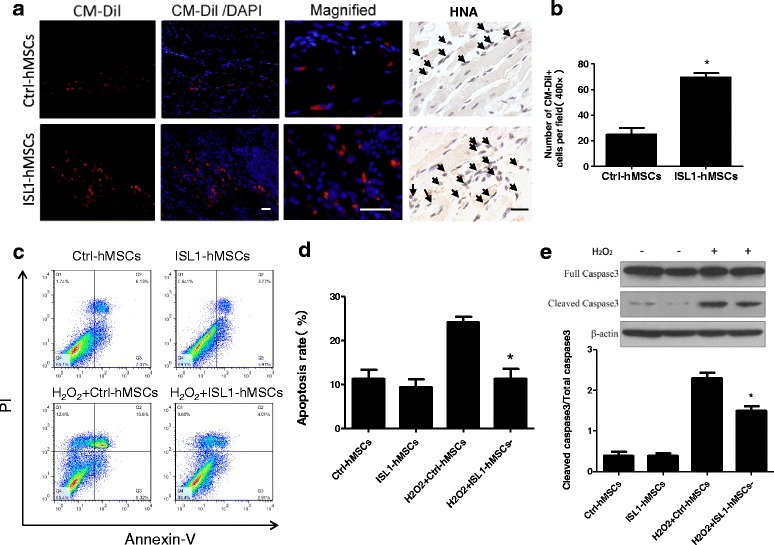
Islet-1 (ISL1) overexpression increased human mesenchymal stem cell (hMSC) survival after transplantation of post-myocardial infection (MI) hearts. Representative images (**a**) and quantification (**b**) of surviving ISL1-hMSCs and control (Ctrl)-hMSCs in hearts at 7 days after transplantation. Red immunofluorescence of CM-Dil indicates surviving cells, and blue color of 4’6-diamidino-2-phenylindole (DAPI) indicates nuclei. Human nuclear antigen (HNA) staining is also shown. Representative flow cytometric images (**c**) with Annexin V/propidium iodide (PI) double staining assay of cells treated with or without H_2_O_2_. Quantification of Annexin-V-positive cells is shown as the apoptosis rate (**d**): (Annexin-V-positive cell amount / total cell amount) × 100%. Representative images and quantification (**e**) of cleaved caspase3 and full caspase3 in ISL1-hMSCs and Ctrl-hMSCs. Data are presented as the mean ± SD, *n* = 3; *p < 0.05 vs. Ctrl-hMSCs or H_2_O_2_ + Ctrl-hMSCs. Scale bar = 50 μm

To quantify the survival of transplanted cells they were stained with CM-Dil before injection. There were significantly more surviving cells in the myocardium in the ISL1-hMSC group compared with the hMSC group 7 days after transplantation (24.8 ± 5.26% vs. 69.4 ± 7.76%, *n* = 3; *p* < 0.05). Human nuclear antigen (HNA) staining was also used to track grafted cells (Fig. 5a, b). The results were consistent with the CM-Dil tracking.

The improved survival rate of ISL1-hMSCs was confirmed by FACS analysis of cells treated with H_2_O_2_. Hypoxia and oxidative stress in the ischemic area of the post-MI heart are thought to be the main causes of the death of resident transplanted hMSCs and cardiomyocytes. H_2_O_2_ was used to treat hMSCs to simulate oxidative stress in vitro. The apoptosis of hMSCs was assessed by FACS analysis of Annexin V. The increase in Annexin V-positive cells induced by H_2_O_2_ was significantly blunted by ISL1 overexpression (Fig. 5c, d). Western blotting showed that the increases in cleaved caspase3/total caspase3 in hMSCs were partly reversed by ISL1 overexpression (Fig. 5e). Bax, Bcl-2, cleaved caspase 9, and total caspase 9 were also detected by Western blotting. The results were consistent with those for caspase 3 (Additional file 2: Figure S7).

### ISL1 overexpression enhanced the secretion of anti-apoptotic hMSC factors to protect cardiomyocytes

**Fig. 6:**
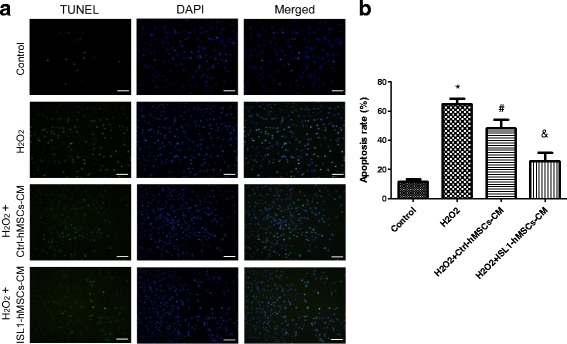
The anti-apoptotic effect of islet-1 human mesenchymal stem cells (ISL1-hMSCs) conditioned medium (CM) on cardiomyocyte cell line H9c2 subjected to oxidative injury (**a**) and quantification (**b**) of TUNEL staining in cardiomyocytes subjected to 200 μM H_2_O_2_ for 6 h. Cell nuclei were stained with 4’6-diamidino-2-phenylindole (DAPI; blue) and TUNEL-positive nuclei (green). Apoptosis rate = (TUNEL-positive nuclei / DAPI-positive nuclei) × 100%. Data are presented as the mean ± SD, n = 3; *p < 0.05 vs. control; ^#^*p* < 0.05 vs. H_2_O_2_; ^&^*p* < 0.05 vs. H_2_O_2_ + Ctrl-hMSCs. Scale bar = 100 μm. Ctrl control

We examined whether conditioned medium from ISL1-overexpressing hMSCs affected the survival of the cardiomyocyte cell line H9c2. H9c2 cells were treated with 200 μM H_2_O_2_ for 6 h. TUNEL-positive cells were detected to determine the apoptotic index. hMSCs-CM decreased TUNEL-positive cells compared with H9c2 cells alone, and ISL1-hMSCs-CM further decreased TUNEL-positive cardiomyocytes. The apoptotic rate was 11.47 ± 0.90% in the normal environment and 64.91 ± 6.07% in oxidative environment. When Ctrl-hMSCs-CM was added, the apoptotic rate decreased to 49.24 ± 7.58%, while in the ISL1-hMSCs-CM group it was 25.80 ± 5.67% (Fig. 6a, b).

### Gene expression profiling of Ctrl-hMSCs and ISL1-hMSCs

**Fig. 7:**
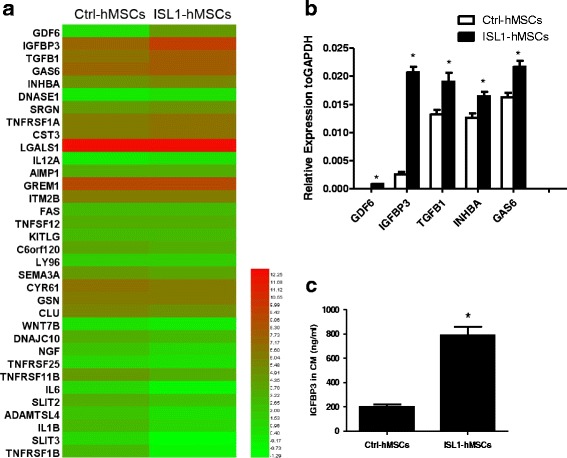
Islet-1 (ISL1) overexpression enhanced the human mesenchymal stem cell (hMSC) paracrine effect by increasing insulin-like growth factor binding protein 3 (IGFBP3) secretion. A heat map display of apoptosis-related genes between ISL1-hMSCs and control (Ctrl)-hMSCs (**a**). GDF6, IGFBP3, TGFB1, GAS6, and INHBA gene expression in Ctrl-hMSCs and ISL1-hMSCs were determined by qPCR (**b**). IGFBP3 in Ctrl-hMSC conditioned medium (CM) and ISL1-hMSCs-CM determined by ELISA assay (**c**). Data are presented as the mean ± SD, *n* = 3; *p < 0.05 vs. Ctrl-hMSCs. GAPDH glyceraldehyde-3-phosphate dehydrogenase

To clarify the molecular basis of the anti-apoptotic effect, gene expression related to variable biological activities between Ctrl-hMSCs and ISL1-hMSCs was detected by RNA-seq (accession number: PRJNA421054). A global overview of the RNA sequencing data is shown in Additional file 1: Tables S2 and S3. The top 10 GO functions of upregulated and downregulated genes of ISL1-MSCs are shown in Additional file 2: Figure S8. Secreted proteins with a RPKM value exceeding 100 in ISL1-hMSCs and Ctrl-hMSCs are shown in Additional file 2: Figure S9. Apoptosis-related secreted factors induced by Ctrl-hMSCs and ISL1-hMSCs are shown in Additional file 1: Table S4. Analysis of apoptosis-related genes showed a higher fold-change in GDF6, IGFBP3, TGFB1, GAS6, and INHBA, among other genes, in ISL1-hMSCs compared with Ctrl-hMSCs (Fig. 7a). To confirm our RNA-Seq results, the expression of the top five apoptosis-related genes with the highest fold-changes (including GDF6, IGFBP3, TGFB1, GAS6, and INHBA) were detected by qPCR in the other three pairs of hMSCs. The results showed that the expression of all five ISL1-hMSC genes was significantly higher than in Ctrl-hMSCs, in which the difference in IGFBP3 was the most prominent (Fig. 7b). We then selected genes by the RPKM value and difference ratio between Ctrl-hMSCs and ISL1-hMSCs. We found that IGFBP3 was the only gene with a RPKM value greater than 100 in ISL1-hMSCs, and the difference ratio was greater than 3, which suggested that IGFBP3 paracrine proteins might play an important role in the anti-apoptosis effect of ISL1-hMSCs. To test this hypothesis, we detected the expression of IGFBP3 in the conditioned medium of ISL1-hMSCs and found that it was approximately three times greater than that of Ctrl-hMSCs (787.89 ± 68.13 vs.198.64 ± 20.30 ng/ml; Fig. 7c).

### IGFBP3 in ISL1-hMSCs-CM had an anti-apoptotic effect on cardiomyocytes subjected to oxidative injury

**Fig. 8:**
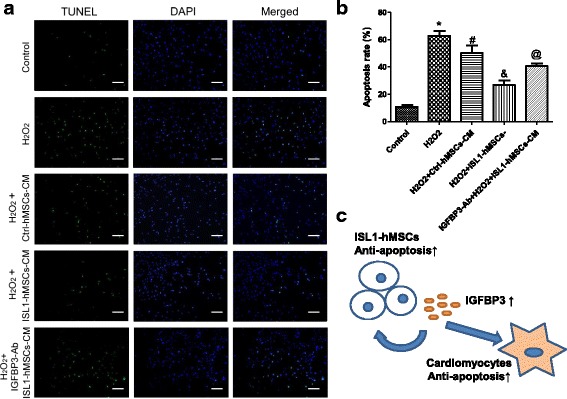
The anti-apoptotic effect of insulin-like growth factor binding protein 3 (IGFBP3) in islet-1 human mesenchymal stem cells (ISL1-hMSCs) conditioned medium (CM) on cardiomyocytes subjected to oxidative injury. Representative images of TUNEL staining in cardiomyocytes (**a**). Cell nuclei were stained with 4’6-diamidino-2-phenylindole (DAPI; blue) and TUNEL-positive nuclei (green). Quantification of TUNEL staining was shown. Apoptosis rate = (TUNEL positive nuclei / DAPI + nuclei) × 100% (**b**). Graphic of the mechanisms underlying the therapeutic effects of transplantation of ISL1-hMSCs (**c**). Data are presented as the mean ± SD, *n* = 3; *p < 0.05 vs. control; ^#^*p* < 0.05 vs. H_2_O_2_; ^&^*p* < 0.05 vs. H_2_O_2_ + Ctrl-hMSCs; ^@^*p* < 0.05 vs. H_2_O_2_ + ISL1-hMSCs. Scale bar =100 μm. Ctrl control

Treatment of H9c2 cells with 200 μM H_2_O_2_ for 6 h caused apoptosis. hMSCs-CM decreased the number of TUNEL-positive cells, and ISL1-hMSCs-CM further decreased the number of TUNEL-positive cardiomyocytes. IGFBP3 neutralization antibody (IGFBP3-Ab) was used to inhibit the effect of IGFBP3 in CM. IGFBP3-Ab at different concentrations, 0.25, 0.5, 1, 1.5, or 2 μg/mL, was added to the ISL1-hMSCs-CM. IGFBP3-Ab was pre-incubated in CM for 30 min. ISL1-hMSCs-CM was then added. Cells with or without ISL1-hMSCs-CM were subjected to 200 μM H_2_O_2_ for another 6 h. The TUNEL assay was performed to detect the reduction in active IGFBP3 in CM, and 1.5 μg/mL IGFBP3-Ab was the ideal concentration (Additional file 2: Figure S10). When 1.5 μg/mL IGFBP3 neutralization antibody was used, the apoptotic effect of the ISL1-hMSCs-CM was clearly reversed, which confirmed the paracrine effect of IGFBP3 in the conditioned medium of ISL1-hMSCs on the rat cardiomyocyte cell line H9c2 (Fig. 8a, b). Similar effects were observed in the human cardiomyocyte cell line AC16 (Additional file 2: Figure S11). Overall, transplantation of hMSCs modified with ISL1 resulted in significantly greater cardiac functional improvement than transplantation of Ctrl-hMSCs in an MI model. Overexpression of ISL1 increased the survival of the transplanted hMSCs in an ischemic environment. Moreover, ISL1-hMSCs reduced the apoptosis of cardiomyocytes. A schematic of the effects of ISL1 on hMSCs and cardiomyocytes is shown (Fig. 8c). Secretion of IGFBP3 increased the survival of hMSCs and cardiomyocytes and improved heart ischemia.

## Discussion

Stem cell therapy to repair damaged myocardium has evolved into a promising treatment for ischemic heart disease. One of the limitations of stem cell therapy for heart disease is the low survival rate of transplanted stem cells [15]. Apoptosis has been implicated as the main contributor to the massive loss of donor cells [16]. The major factor contributing to transplanted cell death appears to be the limited blood supply in the infarct zone that produces hypoxia and oxidative stress. The present study explored a novel approach to enhance the viability of stem cells injected into this harsh environment. We tested a novel hypothesis that overexpression of ISL1 in hMSCs would enhance their survival and protect their paracrine function when injected into the hostile environment of a post-MI heart. Transplantation of modified ISL1-hMSCs reduced the infarct size and improved cardiac function in a murine MI model. The greater potential of ISL1-hMSCs to reduce myocardial damage may be attributable to increased survival after transplantation. We observed that ISL1-hMSCs reduced H_2_O_2_-induced apoptosis in vitro. ISL1-hMSCs-CM also decreased the apoptotic effect of H_2_O_2_ in the cardiomyocyte cell line H9c2. Our study provides strong support for approaches that enhance the survival and function of stem cells injected into hearts with a limited blood supply.

ISL1 has been shown to play an essential role in heart embryogenesis, and ISL1-positive cells have been demonstrated to be cardiovascular progenitors [17, 18]. Although a recent study demonstrated that overexpression of ISL1 in MSCs induced cardiomyocyte differentiation [19], a prior study conducted by our group obtained different results [10]. In our previous study, we found that ISL1 overexpression did not affect their ability to differentiate into cardiomyocytes or endotheliocytes. Further studies suggest that overexpression of ISL1 in hMSCs promotes angiogenesis in vitro and in vivo by increasing the secretion of paracrine factors. In the present study, ISL1-hMSCs reduced H_2_O_2_-induced apoptosis in vitro. In addition, ISL1-hMSCs-CM decreased the apoptotic effect of H_2_O_2_ in the cardiomyocyte cell line H9c2. All the aforementioned results suggested that the effects of ISL1-hMSCs were mainly due to a paracrine effect and not cardiomyocyte differentiation [10].

Previous research has suggested that the ISL1 gene is an attractive reparative target for the treatment of myocardial dysfunction. Barzelay et al. [20] reported that ISL1 gives rise to subpopulations of progenitors in bone marrow and spleen and is re-expressed in the spleen and left ventricle following MI. Intramyocardial gene transfer of ISL1 to the border zone of infarcted hearts results in partial salvage of left ventricular function, enhanced vascularization, and reduced myocardial fibrosis [20]. Li et al. reported that ISL1^+^ cardiac progenitor cells (CPCs), when combined with a suitable vehicle, can produce notable therapeutic effects in the infarcted heart [21]. However, whether the role of ISL1 in recovery after MI is related to an anti-apoptosis effect remains unknown. In our study, ISL1-hMSCs reduced H_2_O_2_-induced apoptosis in vitro. ISL1-hMSCs-CM decreased the apoptotic effect of H_2_O_2_ on the cardiomyocyte cell line H9c2. Due to ischemia, hypoxia, and oxidative stress following infarction, the ability of transplanted cells to survive is compromised, which severely reduces the therapeutic effect of stem cell transplantation [22, 23]. This is one of the major barriers to cell therapy in the treatment of MI [24]. Therefore, promoting survival through modulation of the cellular microenvironment is a promising approach for successful stem cell therapy [25]. Our results showed an increased number of surviving cells after transplantation. ISL1-hMSCs also reduced H_2_O_2_-induced apoptosis in vitro. ISL1-hMSCs-CM decreased the apoptotic effect of H_2_O_2_ in the cardiomyocyte cell line H9c2. These results suggested that overexpression of ISL1 might be a novel strategy for enhancing the efficacy of stem cell therapy.

IGFBPs are essential for the transport of insulin-like growth factors (IGFs), prolong their half-lives, and regulate the availability of free IGFs for interaction with IGF receptors, thereby modulating the effects of IGFs. Recent studies have provided ample evidence that IGFBPs have unique activities in addition to interactions with the IGF/IGF-IR (insulin resistance) axis [26]. In particular, IGF/IGF-IR-independent actions of IGFBP3 have been shown to contribute to the pathophysiology of a variety of human diseases, such as cancer, diabetes, and Alzheimer’s disease [27]. IGFBP3 regulates the promitogenic and anti-apoptotic functions of IGFs, but it also has independent functions [28]. IGFBP3 can enhance the proliferative effects of IGFs or inhibit IGF actions. In cell culture, IGFBP3 has also been shown to promote both apoptosis and survival. Granata et al. reported that IGFBP3 differentially regulates endothelial cell apoptosis through involvement of the sphingolipid signaling pathways. Moreover, the survival effect of IGFBP3 seems to be mediated by IGF-IR [29]. IGFBP3 positively regulates angiogenesis through involvement of IGF-IR signaling and subsequent sphingomyelin kinase (SphK) activation [30]. IGFBP3 upregulates IGF-1, a well-known anti-apoptotic and proangiogenic factor, activates IGF-IR and its downstream pathways phosphatidyl inositol-3 (PI3K), Akt, and mitogen-activated protein kinase (MAPK) extracellular signal-regulated kinase (ERK)1/2 [31]. However, the effect of IGFBP3 on cardiomyocyte apoptosis is not clear. In this study, we observed the apoptotic-related gene expression profiles of ISL1-hMSCs and found high expression. We also observed a large difference in the fold-change of IGFBP3 between Ctrl-hMSCs and ISL1-hMSCs. The apoptotic effect of ISL1-hMSCs-CM was clearly reversed by the IGFBP3 neutralization antibody. These data suggest that IGFBP3 is an important anti-apoptotic paracrine factor that protects cardiomyocytes in ischemic hearts. We will explore additional molecular signaling mechanisms, such as IGF-IR, SphK activation, PI3K, Akt, or the MAPK signaling pathway in the future.

IGFBP3 has numerous functional roles, many of which are associated with the functional regulation of IGFs (IGF-dependent effects). Other major functional roles are related to their IGF-independent actions. The direct role of IGFBP3 binding is the target of ongoing research. Kazezian et al. described interferon-alpha signaling pathway activation with IFIT3 and IGFBP3 upregulation, which may affect cellular function in human degenerative discs [32]. IGFBP3 has been identified as the most strongly upregulated gene in degenerative human annulus fibrosis. The interferon-alpha signaling pathway is activated in the human degenerative annulus fibrosis via induction of 3 IFITs and other genes, such as IGFBP3. In contrast, He et al. found that activation of IGF-1/IGFBP3 signaling by berberine improves the intestinal mucosal barrier in rats with acute endotoxemia [33]. Deng et al. reported that IGFBP3 is an important MSC homing molecule and reported on the therapeutic potential of hMSC extracellular matrix in bone regeneration [34]. In our study, overexpression of ISL1 in hMSCs promoted cell survival in a model of MI and enhanced their paracrine functions to protect cardiomyocytes, which might be completed through IGFBP3. Our study provides new experimental evidence for the research of IGFBP3.

## Conclusions

Overall, this study suggests that overexpression of ISL1 in hMSCs promoted cell survival in a model of MI and enhanced their paracrine function to protect cardiomyocytes, which may be mediated through IGFBP3. Overexpression of ISL1 could be a novel strategy for enhancing the efficacy of stem cell-mediated cardiac repair after MI.

## Additional files

Additional file 1: **Table S1.** qPCR primer information. **Table S2.** Sequencing data statistical results. **Table S3.** Alignment data statistical results. **Table S4.** Apoptosis-related secreted factors induced by Ctrl-hMSCs and ISL1-hMSCs. (PPTX 81 kb)

Additional file 2: **Figure S1.** Transplantation of ISL1-hMSCs improved cardiac function (LVEDV, LVESV and CO) in amyocardial infarction (MI) model. ^*^p < 0.05 vs. sham; ^#^*p* < 0.05 vs. MI + PBS; ^& ^*p* < 0.05 vs. MI + ISL1-hMSCs. **Figure S2.** ISL1 overexpression reduced TUNEL-positive cardiomyocytes in infarct hearts. Scale bar = 50 μm. **Figure S3.** ISL1 overexpression reduced CD3+ T lymphocytes in infarct hearts. Scale bar = 50 μm. **Figure S4.** ISL1 overexpression reduced the number of CD68+ T lymphocytes in infarct hearts. Scale bar = 50 μm. **Figure S5.** ISL1 overexpression reduced inflammation cytokines TNFα, IL-6, and IL-10. Scale bar = 50 μm. **Figure S6.** ISL1 overexpression downregulated the proliferation and proinflammatory cytokine production of CD3+ T cells in vitro. ^*^p < 0.05 vs. control; ^#^*p* < 0.05 vs. Ctrl-hMSCs. **Figure S7.** Representative images and quantification of Bax, Bcl-2, cleaved caspase 3, and full-length caspase 3 in ISL1-hMSCs and Ctrl-hMSCs with or without H2O2. ^*^p < 0.05 vs. H2O2 + Ctrl-hMSCs. **Figure S8.** Top 10 GO functions of upregulated (a) and downregulated (b) genes in ISL1-MSCs. **Figure S9.** Heat map display of secreted proteins with RPKM values of more than 100 in ISL1-hMSCs and Ctrl-hMSCs. **Figure S10.** The IGFBP3 inhibition assay showed a reduction in active IGFBP3 in ISL1-hMSCs-CM. ^*^p < 0.05 vs. control; ^#^*p* < 0.05 vs. H2O2; ^&^*p* < 0.05 vs. H2O2 + ISL1-hMSCs. Scale bar = 100 μm. **Figure S11.** The anti-apoptotic effect of IGFBP3 in ISL1-hMSCs-CM on the human cardiomyocyte cell line AC16 subjected to oxidative injury. Apoptosis rate = (TUNEL positive nuclei / DAPI + nuclei) × 100%. ^*^p < 0.05 vs. control; ^#^*p* < 0.05 vs. H2O2; ^&^*p* < 0.05 vs. H2O2 + Ctrl-hMSCs; ^@^*p* < 0.05 vs. H2O2 + ISL1-hMSCs. Scale bar = 100 μm. DAPI: 4′,6- diamidino-2-phenylindole. (PPT 15681 kb)

## Abbreviations

CM: Conditioned medium
CO: Cardiac output
DAPI: 4’6-Diamidino-2-phenylindole
DMEM: Dulbecco’s modified Eagle’s medium
EF: Ejection fraction
EF1α: Elongation factor 1α
ELISA: Enzyme-linked immunosorbent assay
FACS: Fluorescence-activated cell sorting
FBS: Fetal bovine serum
FITC: Fluorescein isothiocyanate
FS: Fractional shortening
GO: Gene Ontology
hMSC: Human mesenchymal stem cell
HNA: Human nuclear antigen
IFN: Interferon
IGF: Insulin-like growth factor
IGFBP3: Insulin-like growth factor binding protein 3
IL: Interleukin
IR: Insulin resistance
ISL1: Islet-1
LAD: Left anterior descending coronary artery
LVEDV: Left ventricular end-diastolic volume
LVESV: Left ventricular end-systolic volume
MI: Myocardial infarction
PBS: Phosphate-buffered saline
PCR: Polymerase chain reaction
PE: Phycoerythrin
PHD2: Prolyl hydroxylase domain protein 2
PI: Propidium iodide
qRT-PCR: Quantitative reverse-transcription polymerase chain reaction
SD: Standard deviation
TGF: Transforming growth factor
TNF: Tumor necrosis factor
TUNEL: Transferase-mediated deoxyuridine triphosphate-biotin nick end labeling
