# Supplementary material for: ISL1 overexpression enhances the survival of transplanted human mesenchymal stem cells in a murine myocardial infarction model
Source: Stem Cell Res Ther. 2018 Feb 26;9:51. doi: 10.1186/s13287-018-0803-7 (PMC5828309; doi:10.1186/s13287-018-0803-7)
Supplement: Supplementary file 1 — Table S1. qPCR primer information. Table S2. Sequencing data statistical results. Table S3. Alignment data statistical results. Table S4. Apoptosis-related secreted factors induced by Ctrl-hMSCs and ISL1-hMSCs. (PPTX 81 kb) [file 13287_2018_803_MOESM1_ESM.pptx]

## Slide 1
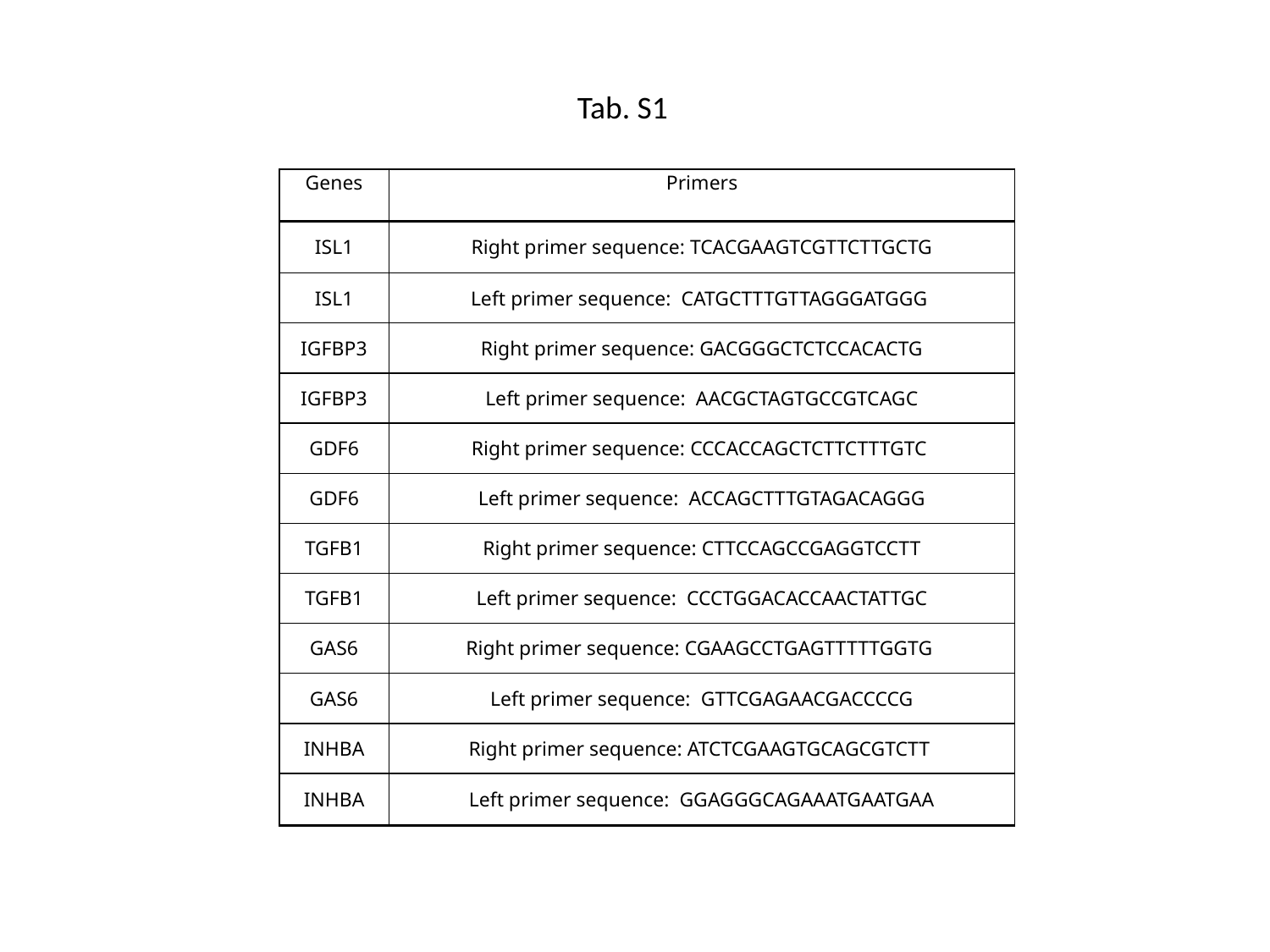

Tab. S1
| Genes | Primers |
| --- | --- |
| ISL1 | Right primer sequence: TCACGAAGTCGTTCTTGCTG |
| ISL1 | Left primer sequence: CATGCTTTGTTAGGGATGGG |
| IGFBP3 | Right primer sequence: GACGGGCTCTCCACACTG |
| IGFBP3 | Left primer sequence: AACGCTAGTGCCGTCAGC |
| GDF6 | Right primer sequence: CCCACCAGCTCTTCTTTGTC |
| GDF6 | Left primer sequence: ACCAGCTTTGTAGACAGGG |
| TGFB1 | Right primer sequence: CTTCCAGCCGAGGTCCTT |
| TGFB1 | Left primer sequence: CCCTGGACACCAACTATTGC |
| GAS6 | Right primer sequence: CGAAGCCTGAGTTTTTGGTG |
| GAS6 | Left primer sequence: GTTCGAGAACGACCCCG |
| INHBA | Right primer sequence: ATCTCGAAGTGCAGCGTCTT |
| INHBA | Left primer sequence: GGAGGGCAGAAATGAATGAA |

## Slide 2
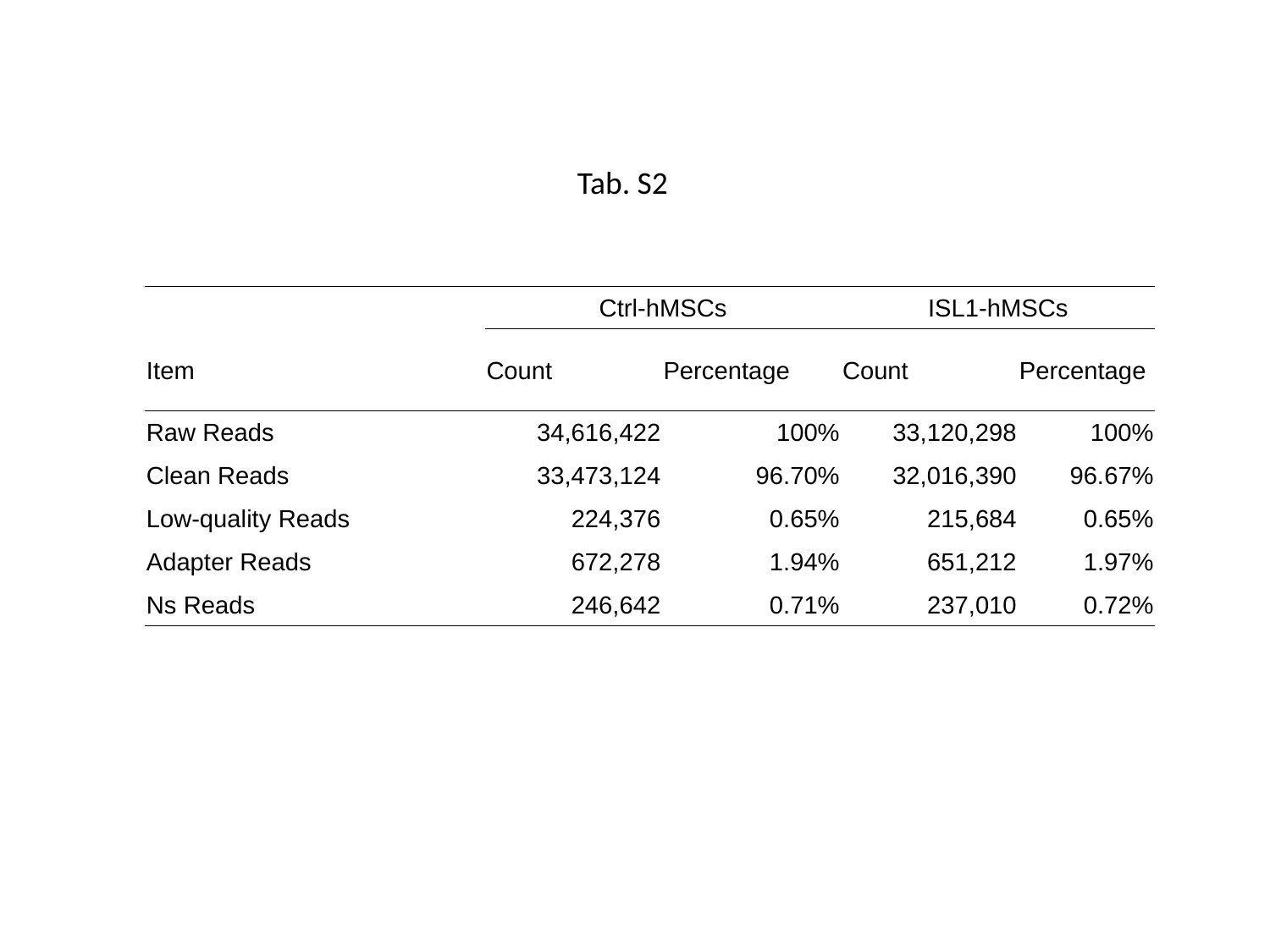

Tab. S2
| | Ctrl-hMSCs | | ISL1-hMSCs | |
| --- | --- | --- | --- | --- |
| Item | Count | Percentage | Count | Percentage |
| Raw Reads | 34,616,422 | 100% | 33,120,298 | 100% |
| Clean Reads | 33,473,124 | 96.70% | 32,016,390 | 96.67% |
| Low-quality Reads | 224,376 | 0.65% | 215,684 | 0.65% |
| Adapter Reads | 672,278 | 1.94% | 651,212 | 1.97% |
| Ns Reads | 246,642 | 0.71% | 237,010 | 0.72% |

## Slide 3
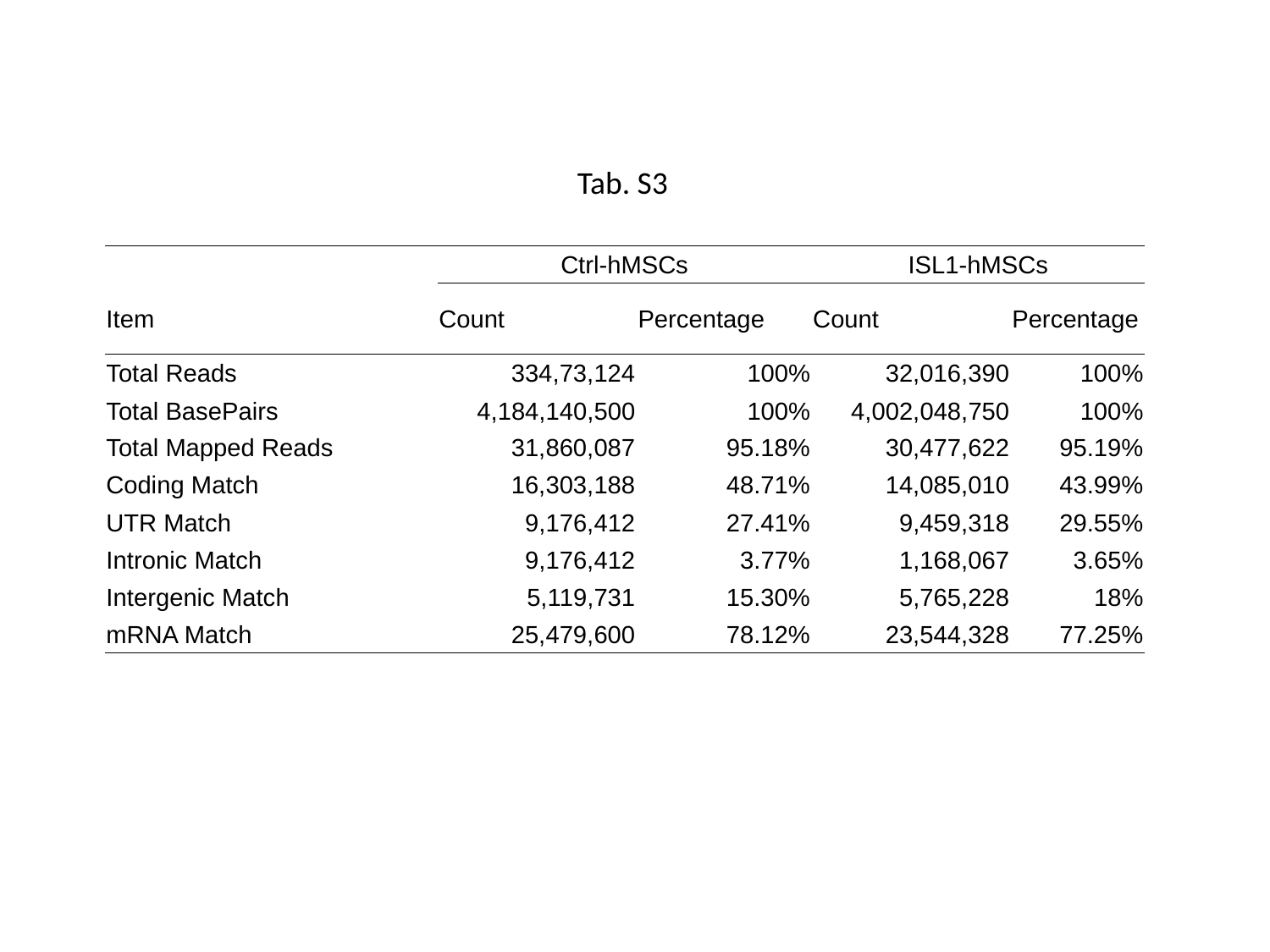

Tab. S3
| | Ctrl-hMSCs | | ISL1-hMSCs | |
| --- | --- | --- | --- | --- |
| Item | Count | Percentage | Count | Percentage |
| Total Reads | 334,73,124 | 100% | 32,016,390 | 100% |
| Total BasePairs | 4,184,140,500 | 100% | 4,002,048,750 | 100% |
| Total Mapped Reads | 31,860,087 | 95.18% | 30,477,622 | 95.19% |
| Coding Match | 16,303,188 | 48.71% | 14,085,010 | 43.99% |
| UTR Match | 9,176,412 | 27.41% | 9,459,318 | 29.55% |
| Intronic Match | 9,176,412 | 3.77% | 1,168,067 | 3.65% |
| Intergenic Match | 5,119,731 | 15.30% | 5,765,228 | 18% |
| mRNA Match | 25,479,600 | 78.12% | 23,544,328 | 77.25% |

## Slide 4
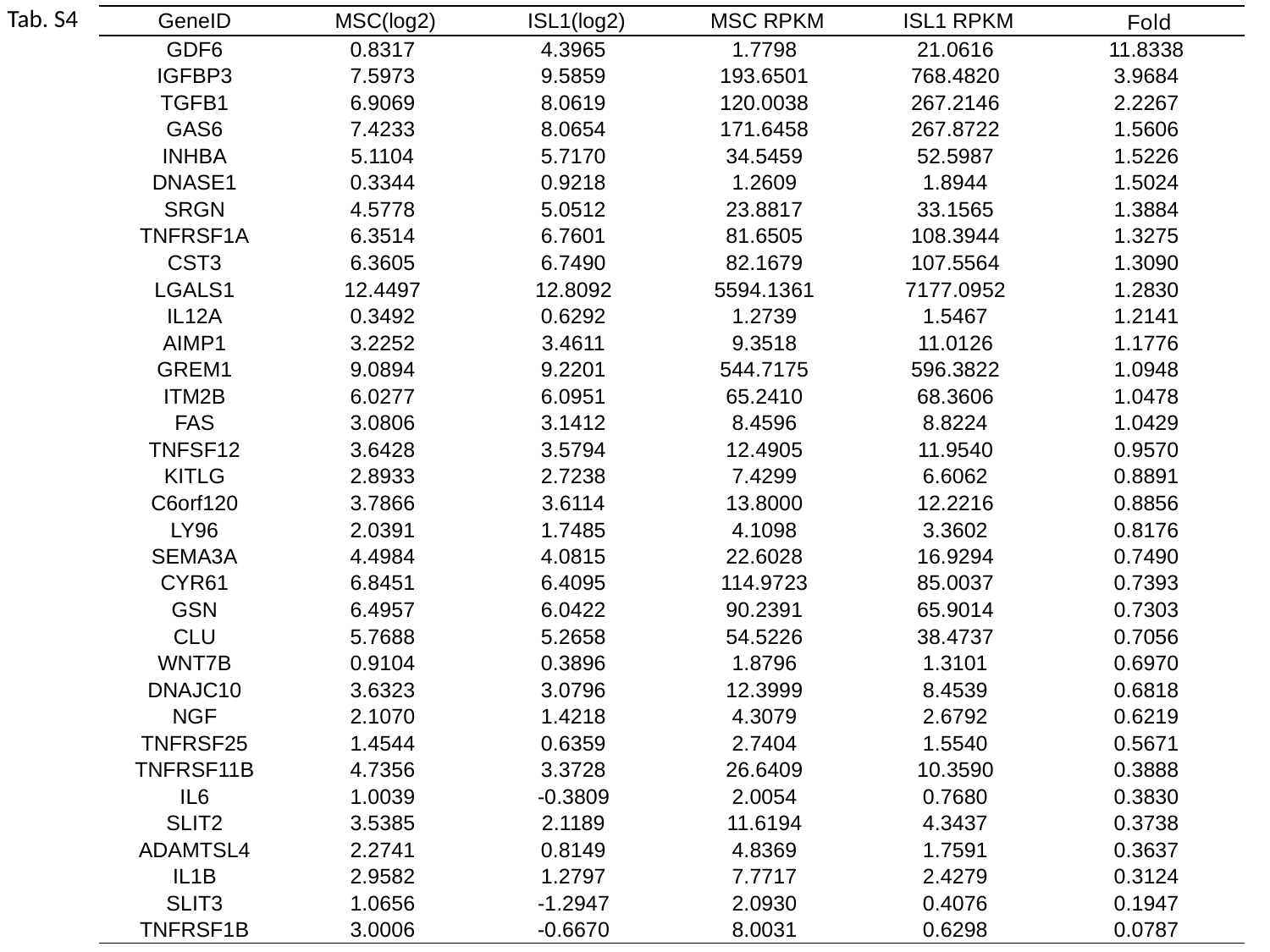

Tab. S4
| GeneID | MSC(log2) | ISL1(log2) | MSC RPKM | ISL1 RPKM | Fold |
| --- | --- | --- | --- | --- | --- |
| GDF6 | 0.8317 | 4.3965 | 1.7798 | 21.0616 | 11.8338 |
| IGFBP3 | 7.5973 | 9.5859 | 193.6501 | 768.4820 | 3.9684 |
| TGFB1 | 6.9069 | 8.0619 | 120.0038 | 267.2146 | 2.2267 |
| GAS6 | 7.4233 | 8.0654 | 171.6458 | 267.8722 | 1.5606 |
| INHBA | 5.1104 | 5.7170 | 34.5459 | 52.5987 | 1.5226 |
| DNASE1 | 0.3344 | 0.9218 | 1.2609 | 1.8944 | 1.5024 |
| SRGN | 4.5778 | 5.0512 | 23.8817 | 33.1565 | 1.3884 |
| TNFRSF1A | 6.3514 | 6.7601 | 81.6505 | 108.3944 | 1.3275 |
| CST3 | 6.3605 | 6.7490 | 82.1679 | 107.5564 | 1.3090 |
| LGALS1 | 12.4497 | 12.8092 | 5594.1361 | 7177.0952 | 1.2830 |
| IL12A | 0.3492 | 0.6292 | 1.2739 | 1.5467 | 1.2141 |
| AIMP1 | 3.2252 | 3.4611 | 9.3518 | 11.0126 | 1.1776 |
| GREM1 | 9.0894 | 9.2201 | 544.7175 | 596.3822 | 1.0948 |
| ITM2B | 6.0277 | 6.0951 | 65.2410 | 68.3606 | 1.0478 |
| FAS | 3.0806 | 3.1412 | 8.4596 | 8.8224 | 1.0429 |
| TNFSF12 | 3.6428 | 3.5794 | 12.4905 | 11.9540 | 0.9570 |
| KITLG | 2.8933 | 2.7238 | 7.4299 | 6.6062 | 0.8891 |
| C6orf120 | 3.7866 | 3.6114 | 13.8000 | 12.2216 | 0.8856 |
| LY96 | 2.0391 | 1.7485 | 4.1098 | 3.3602 | 0.8176 |
| SEMA3A | 4.4984 | 4.0815 | 22.6028 | 16.9294 | 0.7490 |
| CYR61 | 6.8451 | 6.4095 | 114.9723 | 85.0037 | 0.7393 |
| GSN | 6.4957 | 6.0422 | 90.2391 | 65.9014 | 0.7303 |
| CLU | 5.7688 | 5.2658 | 54.5226 | 38.4737 | 0.7056 |
| WNT7B | 0.9104 | 0.3896 | 1.8796 | 1.3101 | 0.6970 |
| DNAJC10 | 3.6323 | 3.0796 | 12.3999 | 8.4539 | 0.6818 |
| NGF | 2.1070 | 1.4218 | 4.3079 | 2.6792 | 0.6219 |
| TNFRSF25 | 1.4544 | 0.6359 | 2.7404 | 1.5540 | 0.5671 |
| TNFRSF11B | 4.7356 | 3.3728 | 26.6409 | 10.3590 | 0.3888 |
| IL6 | 1.0039 | -0.3809 | 2.0054 | 0.7680 | 0.3830 |
| SLIT2 | 3.5385 | 2.1189 | 11.6194 | 4.3437 | 0.3738 |
| ADAMTSL4 | 2.2741 | 0.8149 | 4.8369 | 1.7591 | 0.3637 |
| IL1B | 2.9582 | 1.2797 | 7.7717 | 2.4279 | 0.3124 |
| SLIT3 | 1.0656 | -1.2947 | 2.0930 | 0.4076 | 0.1947 |
| TNFRSF1B | 3.0006 | -0.6670 | 8.0031 | 0.6298 | 0.0787 |
