# Supplementary material for: ISL1 overexpression enhances the survival of transplanted human mesenchymal stem cells in a murine myocardial infarction model
Source: Stem Cell Res Ther. 2018 Feb 26;9:51. doi: 10.1186/s13287-018-0803-7 (PMC5828309; doi:10.1186/s13287-018-0803-7)
Supplement: Supplementary file 2 — Figure S1. Transplantation of ISL1-hMSCs improved cardiac function (LVEDV, LVESV and CO) in amyocardial infarction (MI) model. *p < 0.05 vs. sham; #p < 0.05 vs. MI + PBS; & p < 0.05 vs. MI + ISL1-hMSCs. Figure S2. ISL1 overexpression reduced TUNEL-positive cardiomyocytes in infarct hearts. Scale bar = 50 μm. Figure S3. ISL1 overexpression reduced CD3+ T lymphocytes in infarct hearts. Scale bar = 50 μm. Figure S4. ISL1 overexpression reduced the number of CD68+ T lymphocytes in infarct hearts. Scale bar = 50 μm. Figure S5. ISL1 overexpression reduced inflammation cytokines TNFα, IL-6, and IL-10. Scale bar = 50 μm. Figure S6. ISL1 overexpression downregulated the proliferation and proinflammatory cytokine production of CD3+ T cells in vitro. *p < 0.05 vs. control; #p < 0.05 vs. Ctrl-hMSCs. Figure S7. Representative images and quantification of Bax, Bcl-2, cleaved caspase 3, and full-length caspase 3 in ISL1-hMSCs and Ctrl-hMSCs with or without H2O2. *p < 0.05 vs. H2O2 + Ctrl-hMSCs. Figure S8. Top 10 GO functions of upregulated (a) and downregulated (b) genes in ISL1-MSCs. Figure S9. Heat map display of secreted proteins with RPKM values of more than 100 in ISL1-hMSCs and Ctrl-hMSCs. Figure S10. The IGFBP3 inhibition assay showed a reduction in active IGFBP3 in ISL1-hMSCs-CM. *p < 0.05 vs. control; #p < 0.05 vs. H2O2; &p < 0.05 vs. H2O2 + ISL1-hMSCs. Scale bar = 100 μm. Figure S11. The anti-apoptotic effect of IGFBP3 in ISL1-hMSCs-CM on the human cardiomyocyte cell line AC16 subjected to oxidative injury. Apoptosis rate = (TUNEL positive nuclei / DAPI + nuclei) × 100%. *p < 0.05 vs. control; #p < 0.05 vs. H2O2; &p < 0.05 vs. H2O2 + Ctrl-hMSCs; @p < 0.05 vs. H2O2 + ISL1-hMSCs. Scale bar = 100 μm. DAPI: 4′,6- diamidino-2-phenylindole. (PPT 15681 kb) [file 13287_2018_803_MOESM2_ESM.ppt]

## Slide 1
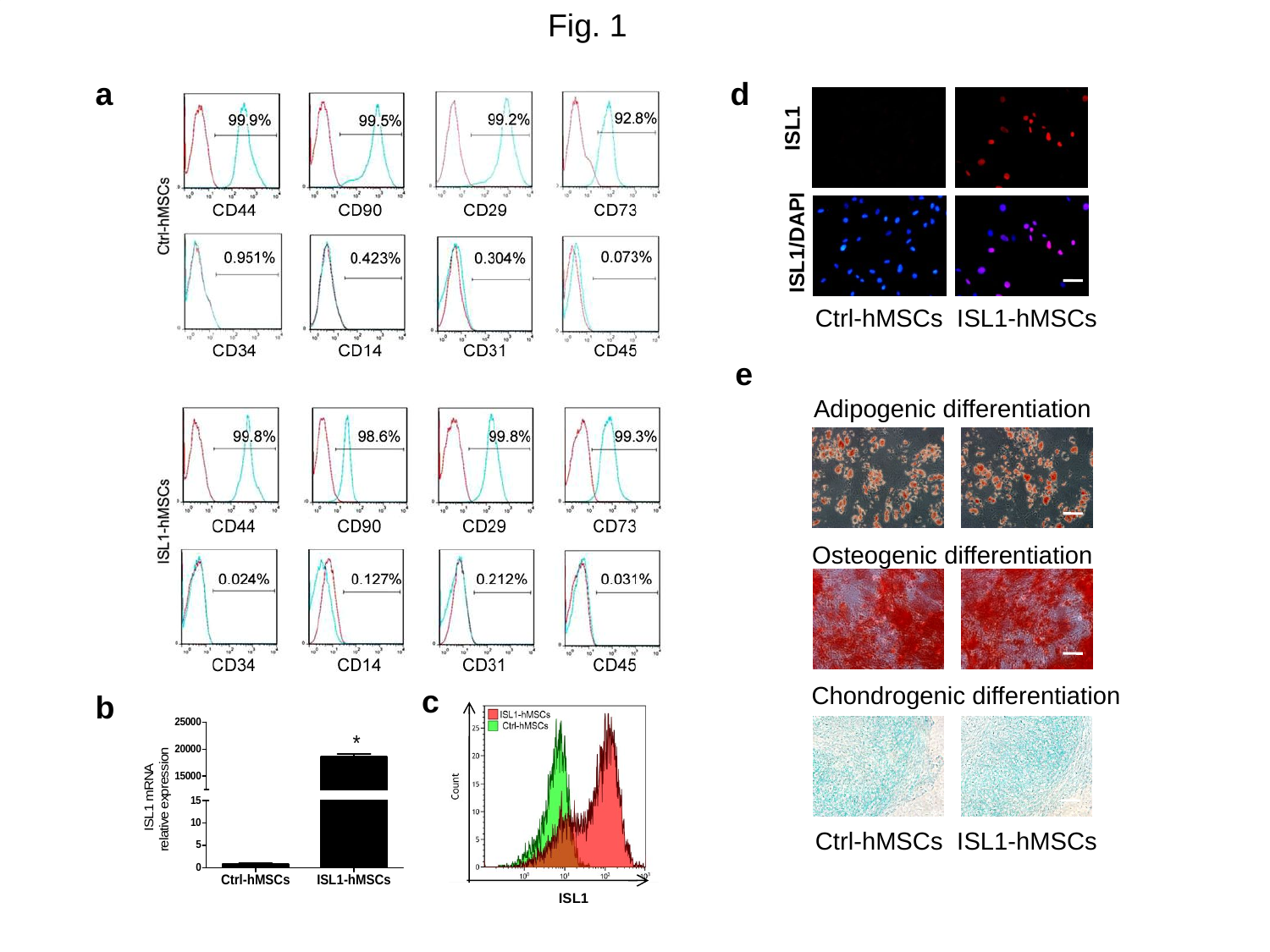

Fig. 1
a
d
ISL1
ISL1/DAPI
Ctrl-hMSCs ISL1-hMSCs
e
Adipogenic differentiation
Osteogenic differentiation
Chondrogenic differentiation
Ctrl-hMSCs ISL1-hMSCs
c
b
ISL1

## Slide 2
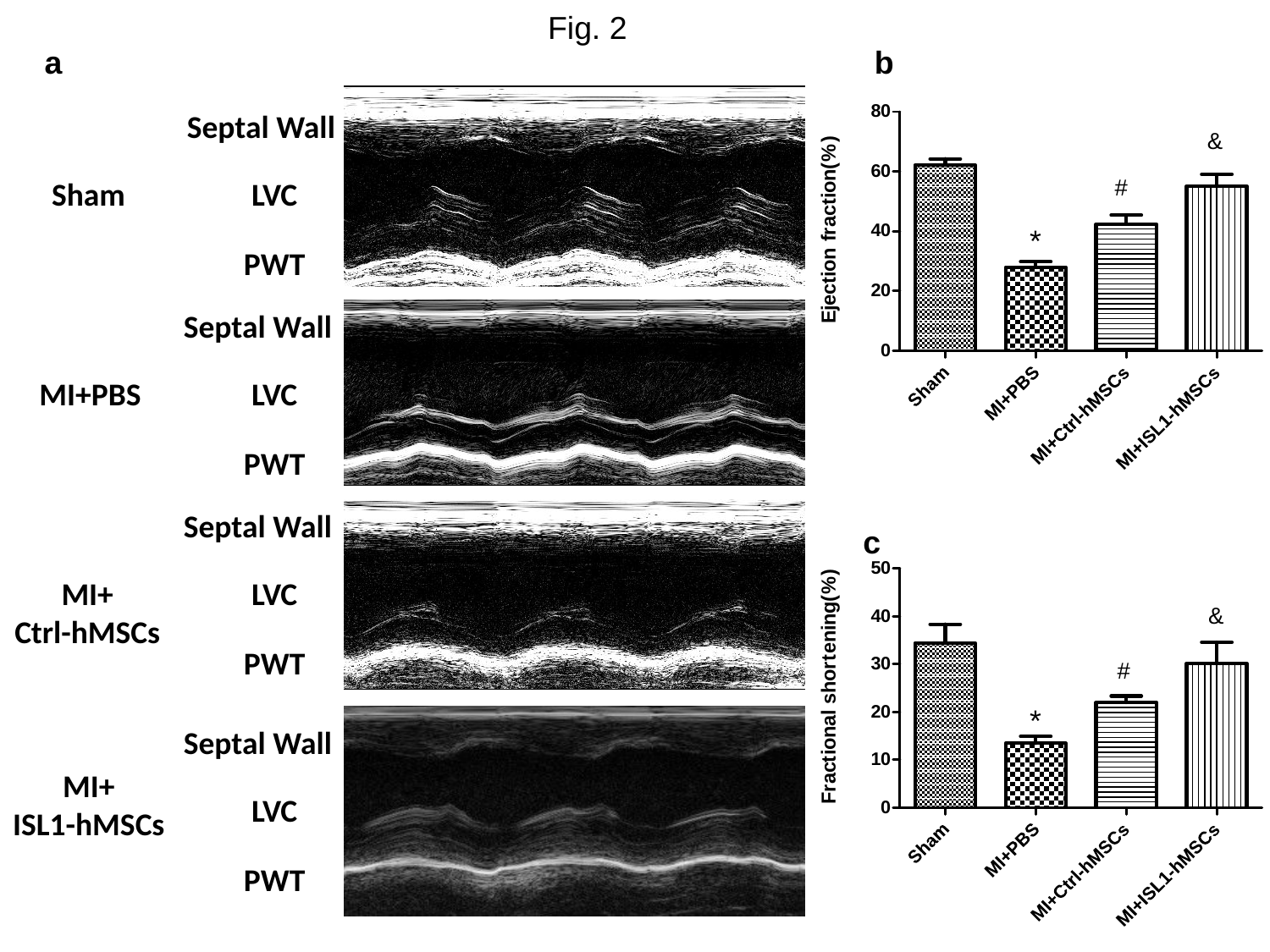

Fig. 2
a
b
Septal Wall
Sham
LVC
PWT
Septal Wall
MI+PBS
LVC
PWT
Septal Wall
MI+
Ctrl-hMSCs
LVC
PWT
Septal Wall
MI+
ISL1-hMSCs
LVC
PWT
c

## Slide 3
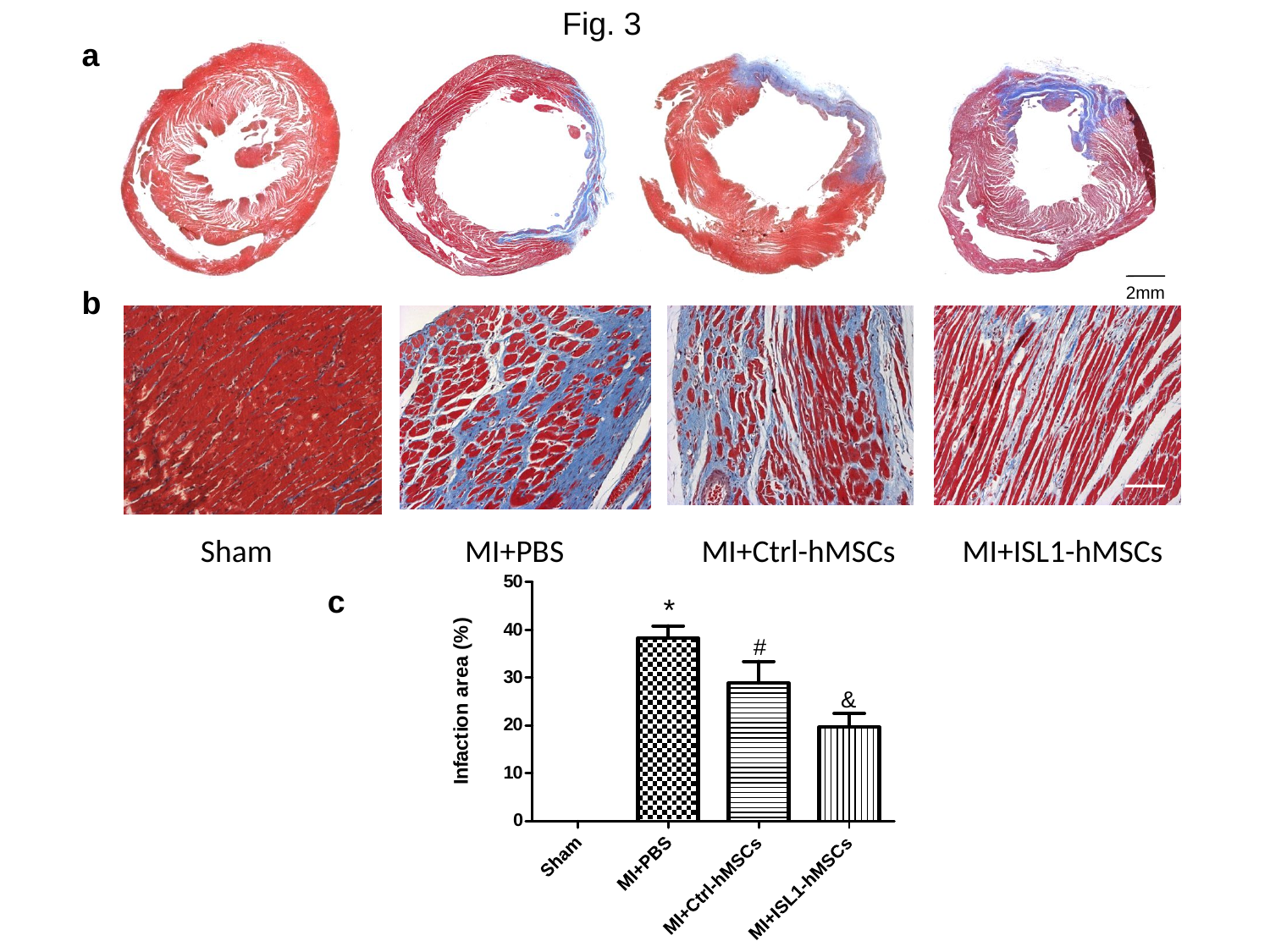

Fig. 3
a
2mm
b
Sham
 MI+PBS
 MI+Ctrl-hMSCs
MI+ISL1-hMSCs
c

## Slide 4
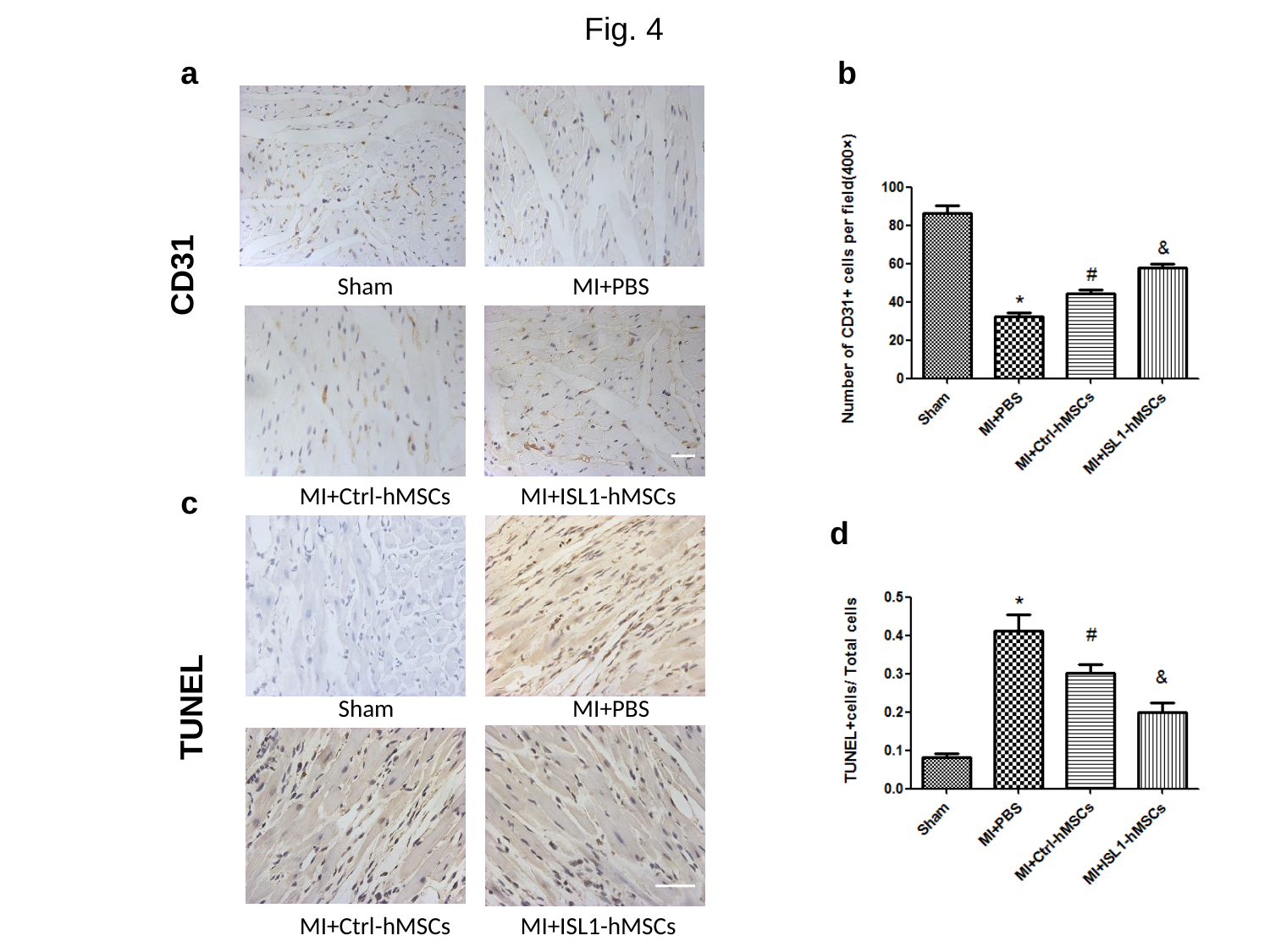

Fig. 4
a
b
CD31
 Sham
 MI+PBS
 MI+Ctrl-hMSCs
MI+ISL1-hMSCs
c
d
TUNEL
 Sham
 MI+PBS
 MI+Ctrl-hMSCs
MI+ISL1-hMSCs

## Slide 5
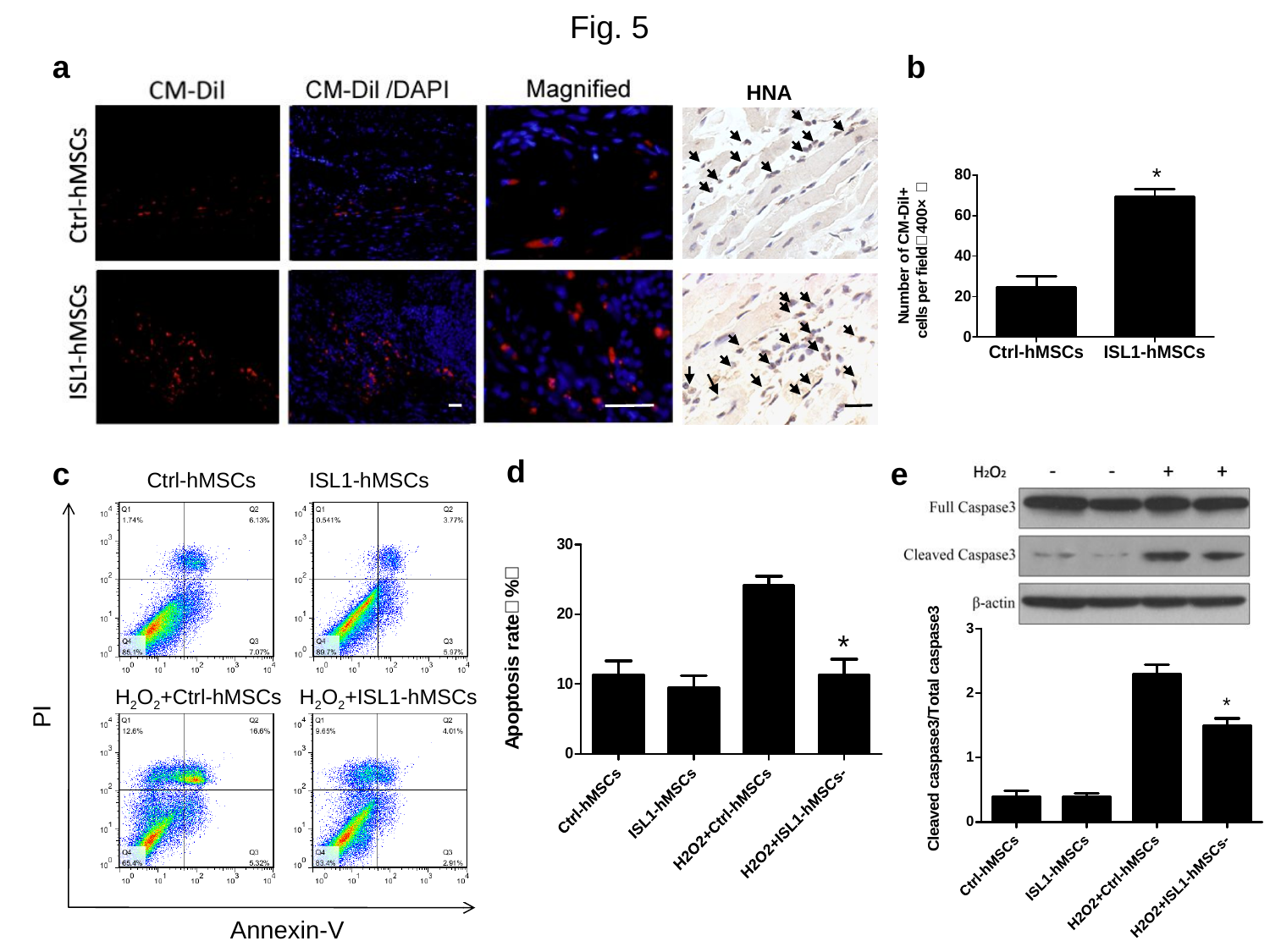

Fig. 5
a
b
HNA
d
c
e
Ctrl-hMSCs ISL1-hMSCs
PI
 H2O2+Ctrl-hMSCs H2O2+ISL1-hMSCs
Annexin-V

## Slide 6
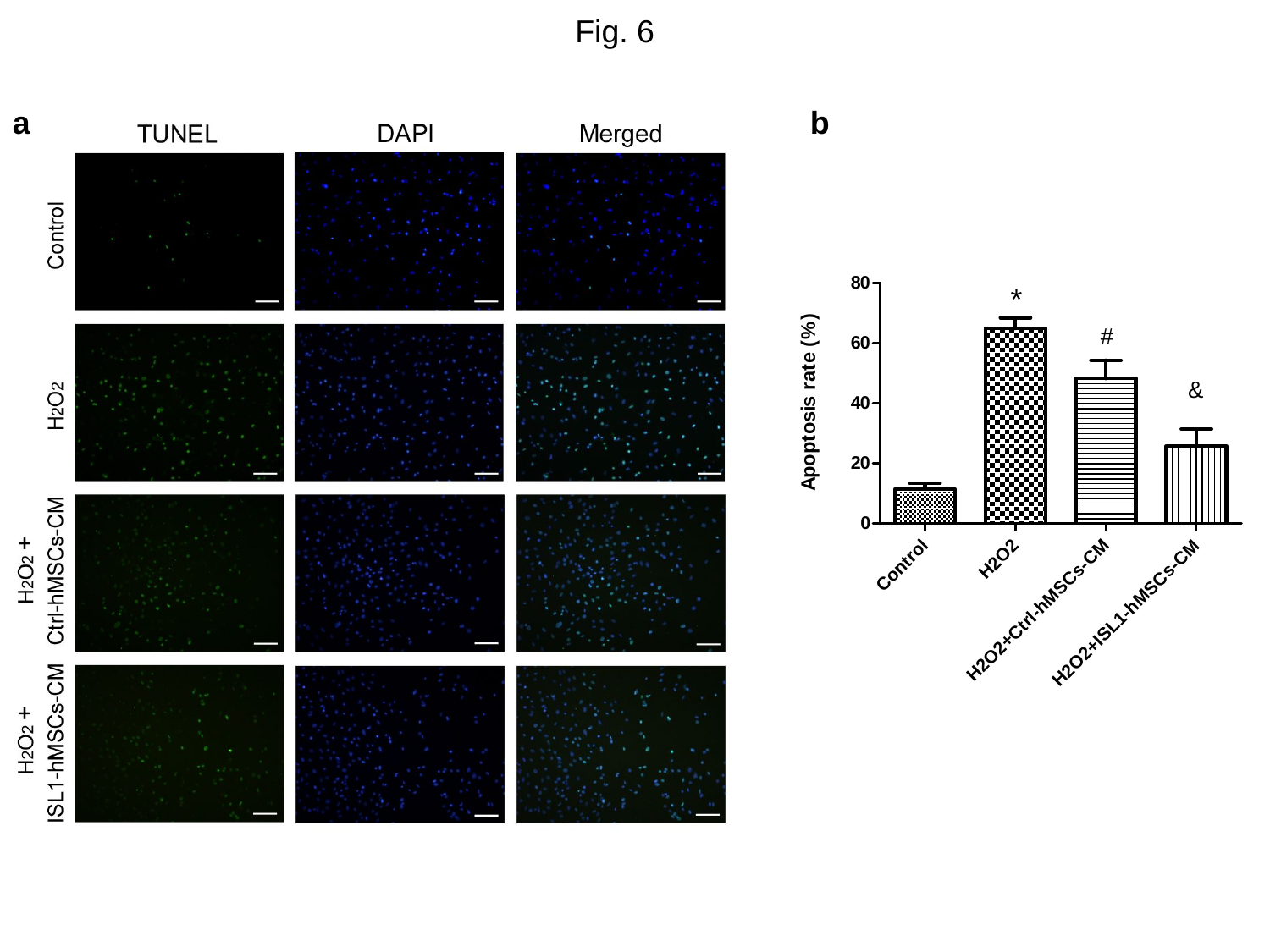

Fig. 6
a
b

## Slide 7
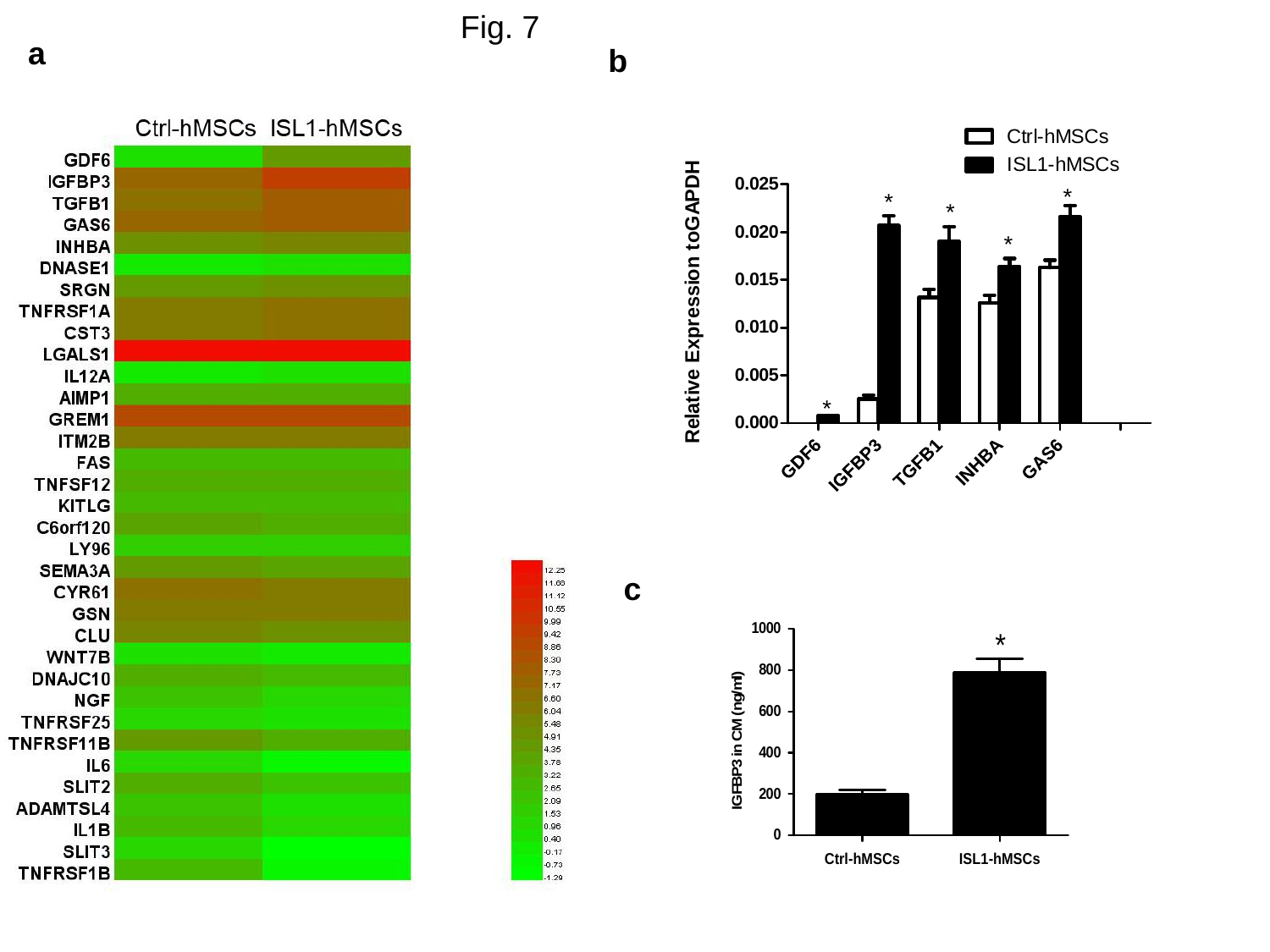

Fig. 7
a
b
c

## Slide 8
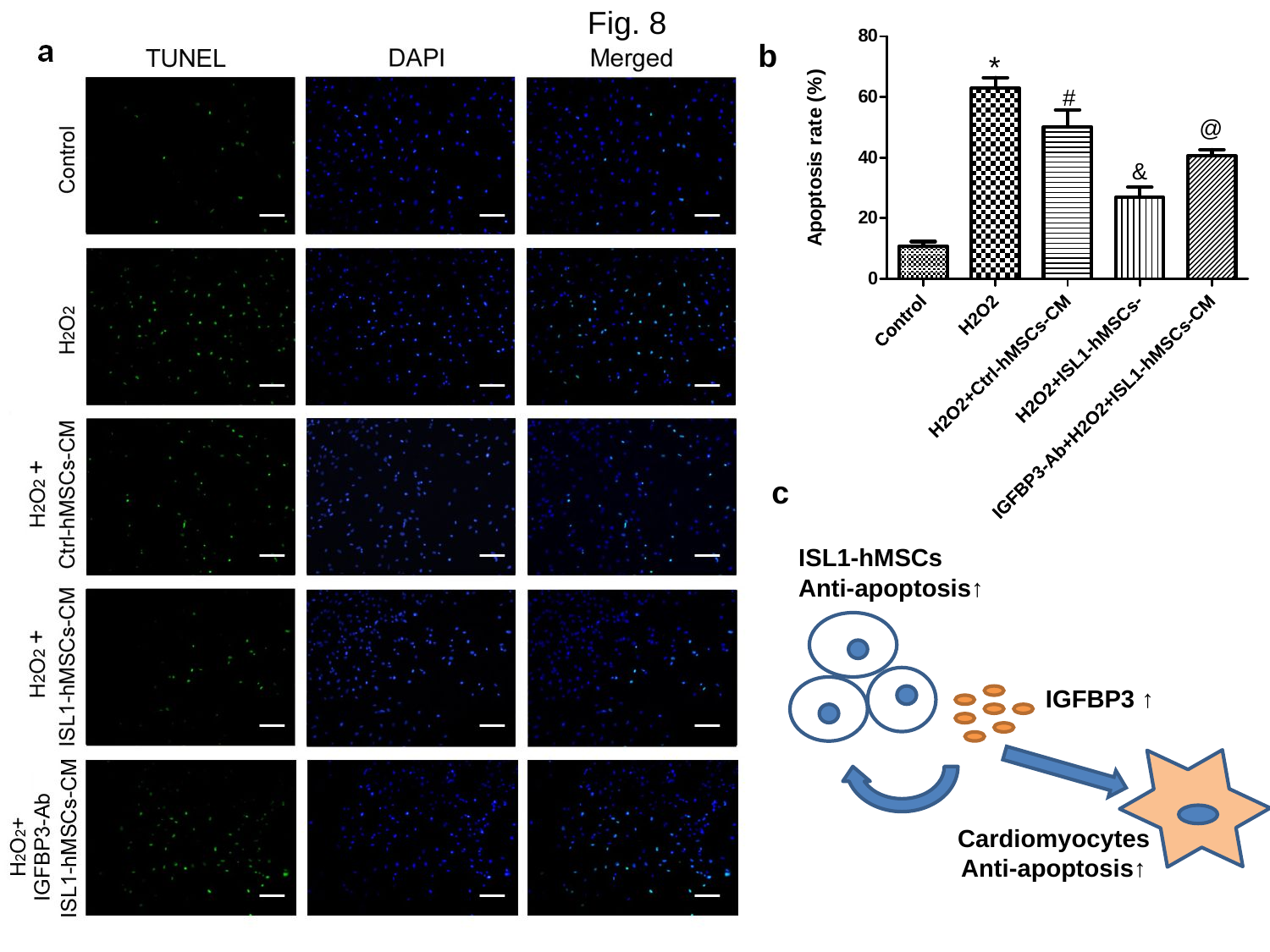

Fig. 8
b
c
ISL1-hMSCs
Anti-apoptosis↑
IGFBP3 ↑
Cardiomyocytes
Anti-apoptosis↑

## Slide 9
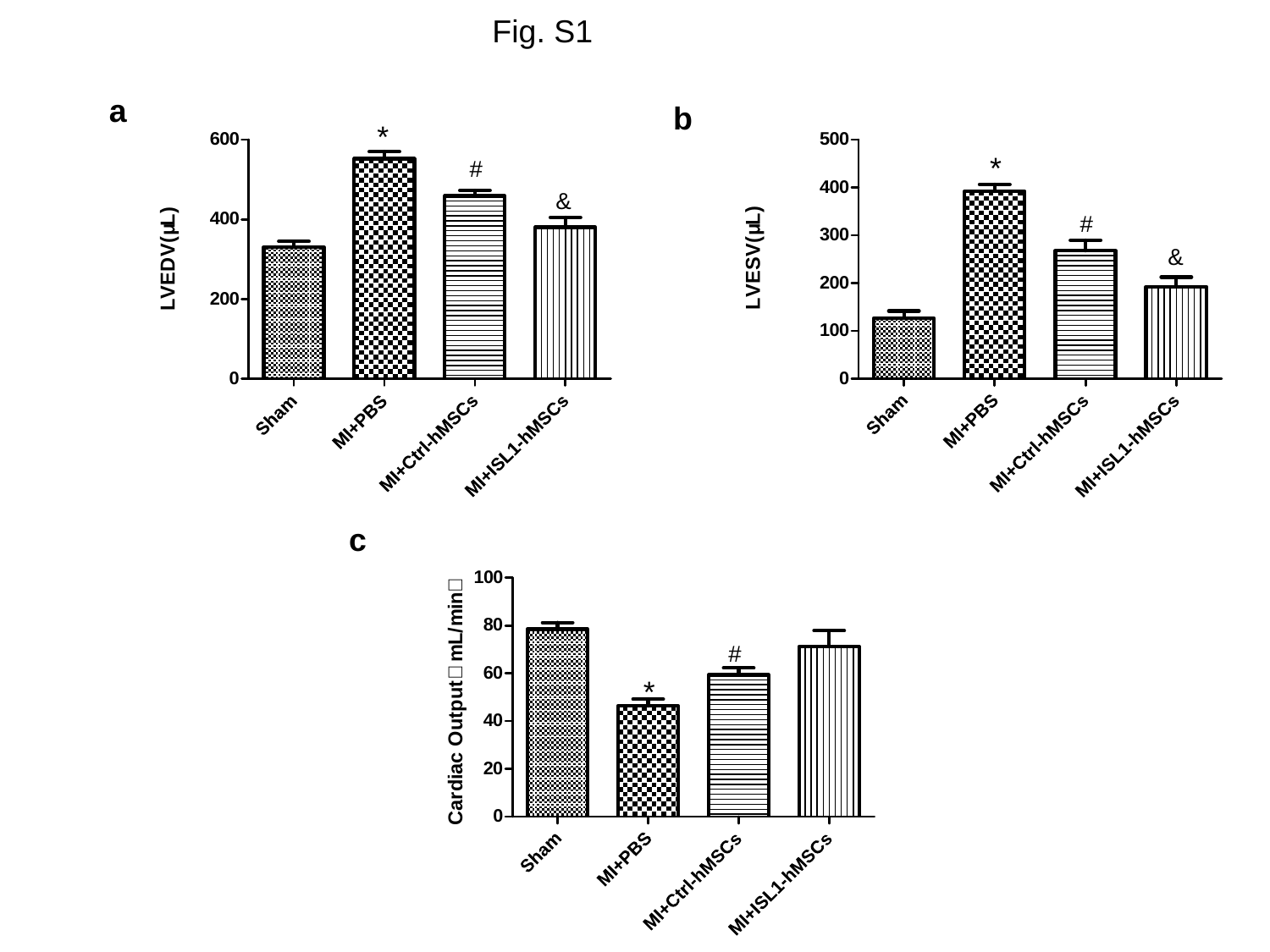

Fig. S1
a
b
c

## Slide 10
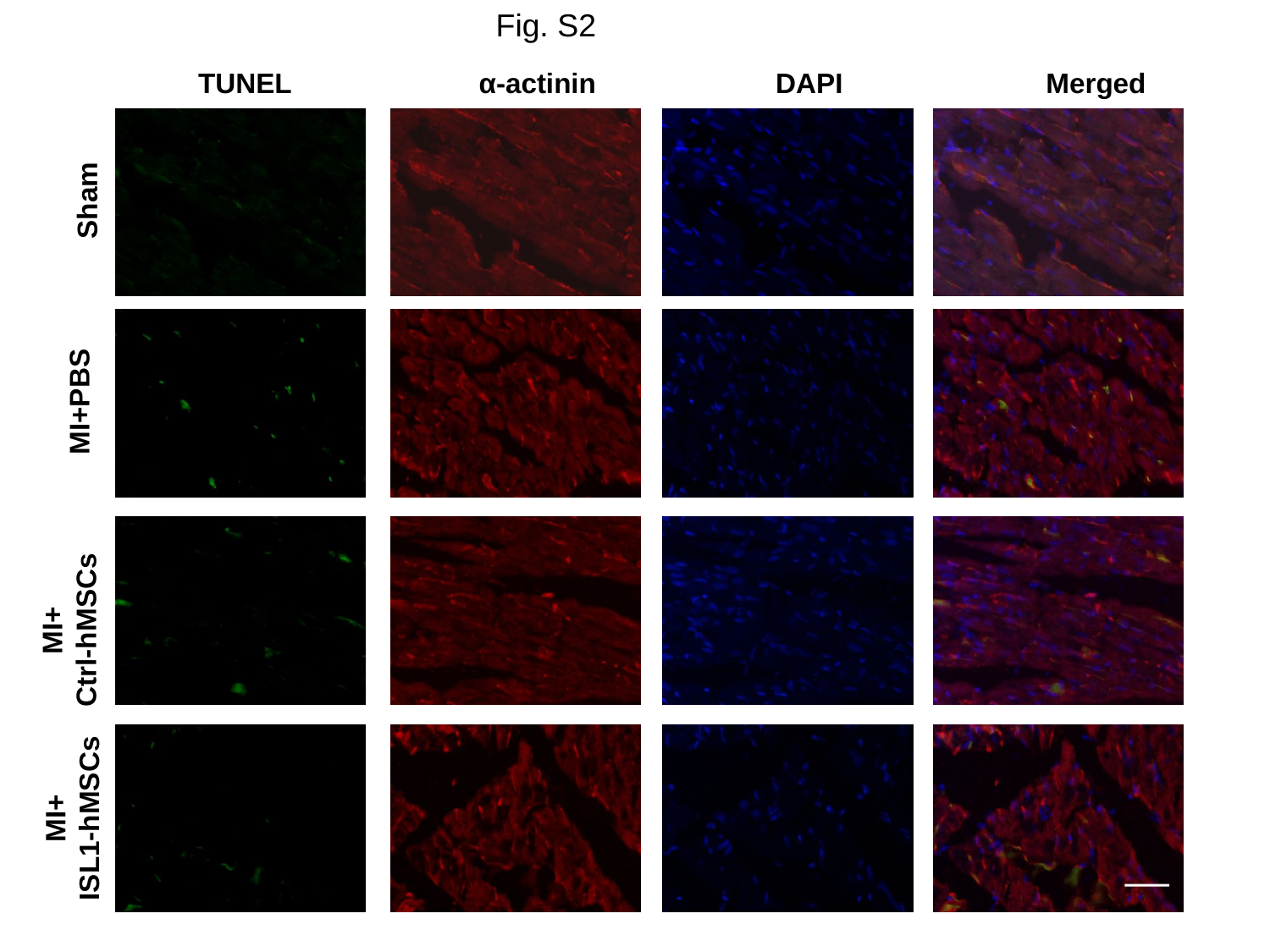

Fig. S2
TUNEL α-actinin DAPI Merged
Sham
MI+PBS
MI+
Ctrl-hMSCs
MI+
ISL1-hMSCs

## Slide 11
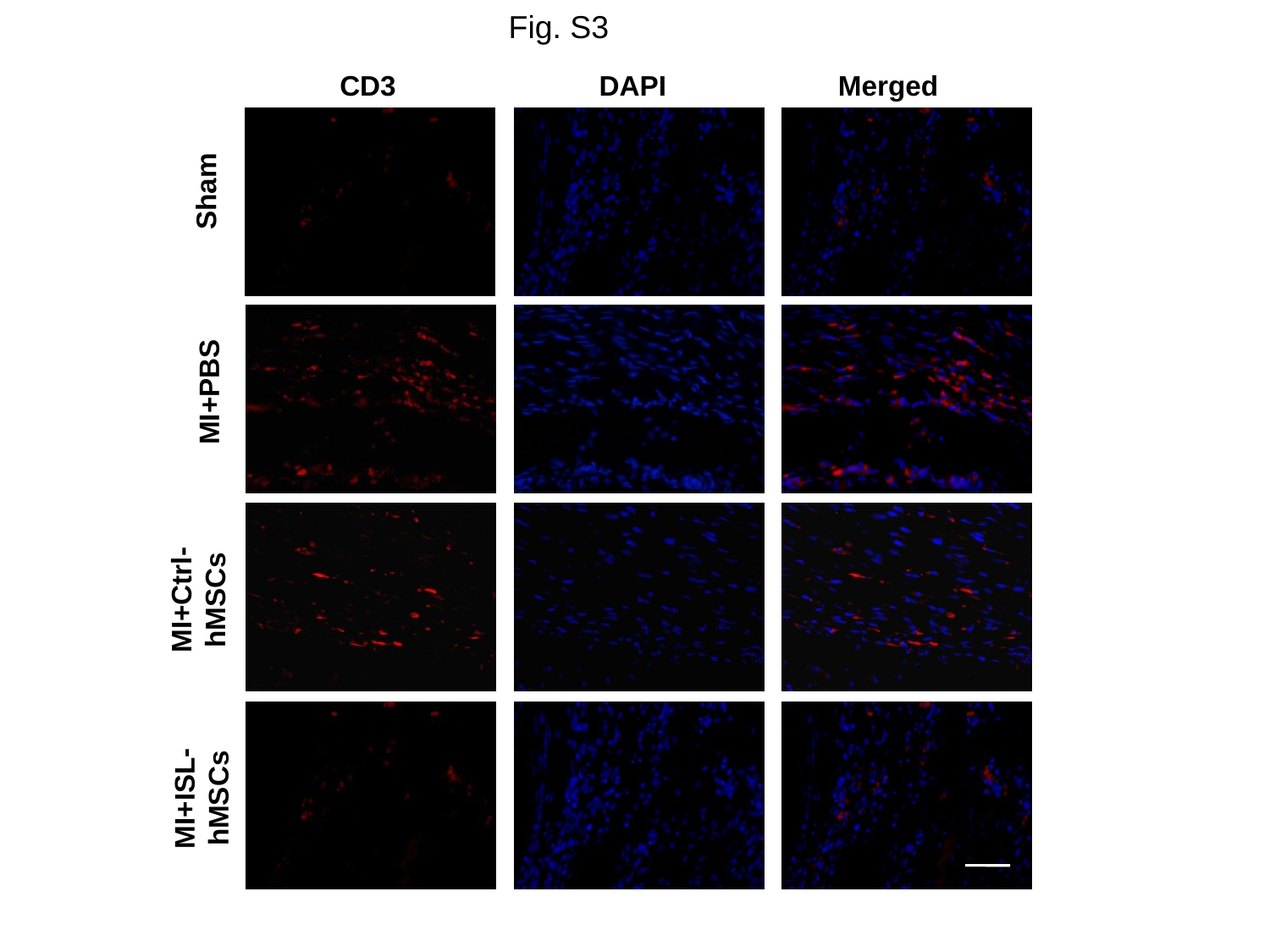

Fig. S3
CD3 DAPI Merged
MI+PBS
MI+Ctrl-hMSCs
MI+ISL-
hMSCs
Sham

## Slide 12
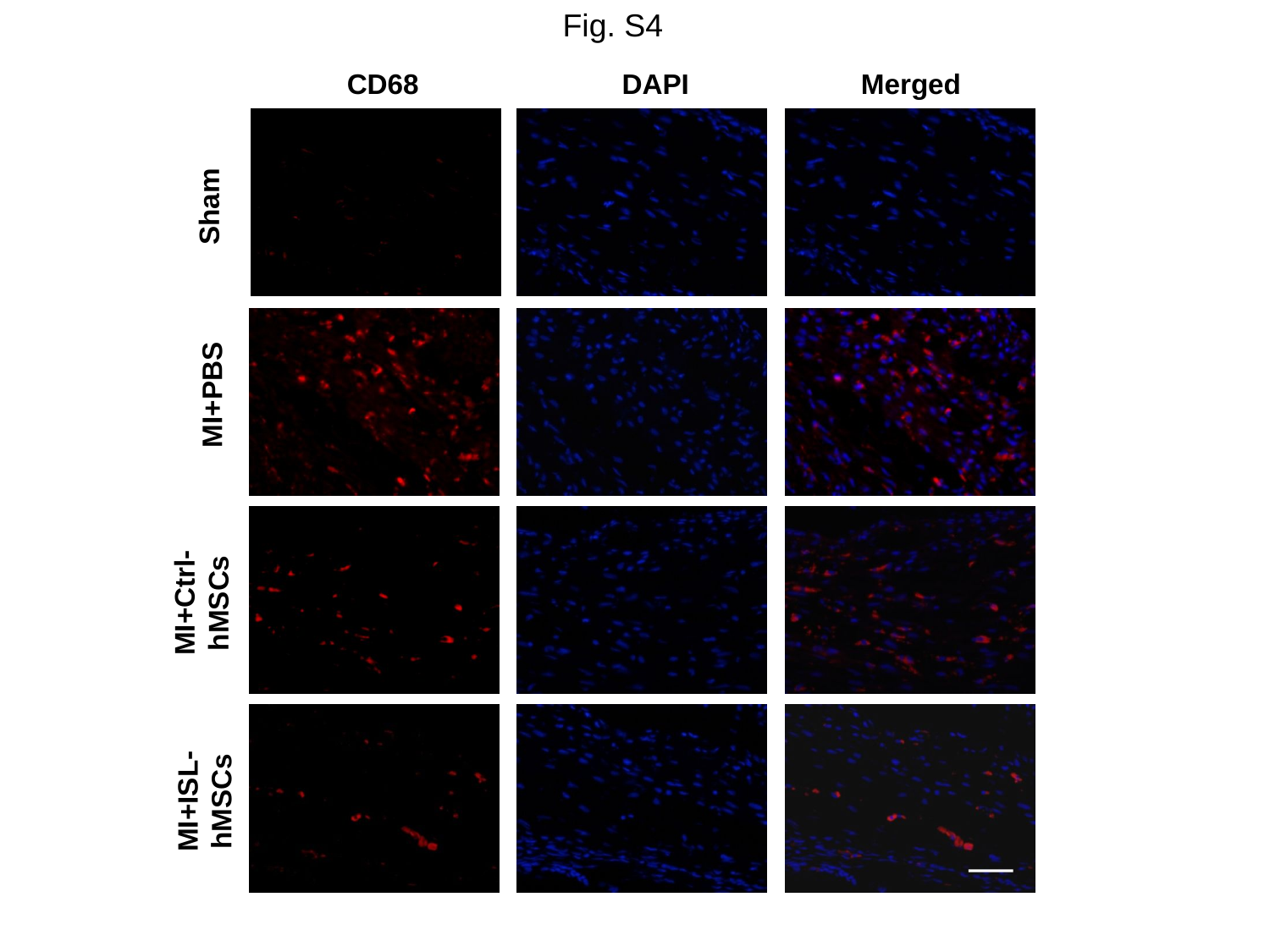

Fig. S4
CD68 DAPI Merged
MI+PBS
MI+Ctrl-hMSCs
MI+ISL-
hMSCs
Sham

## Slide 13
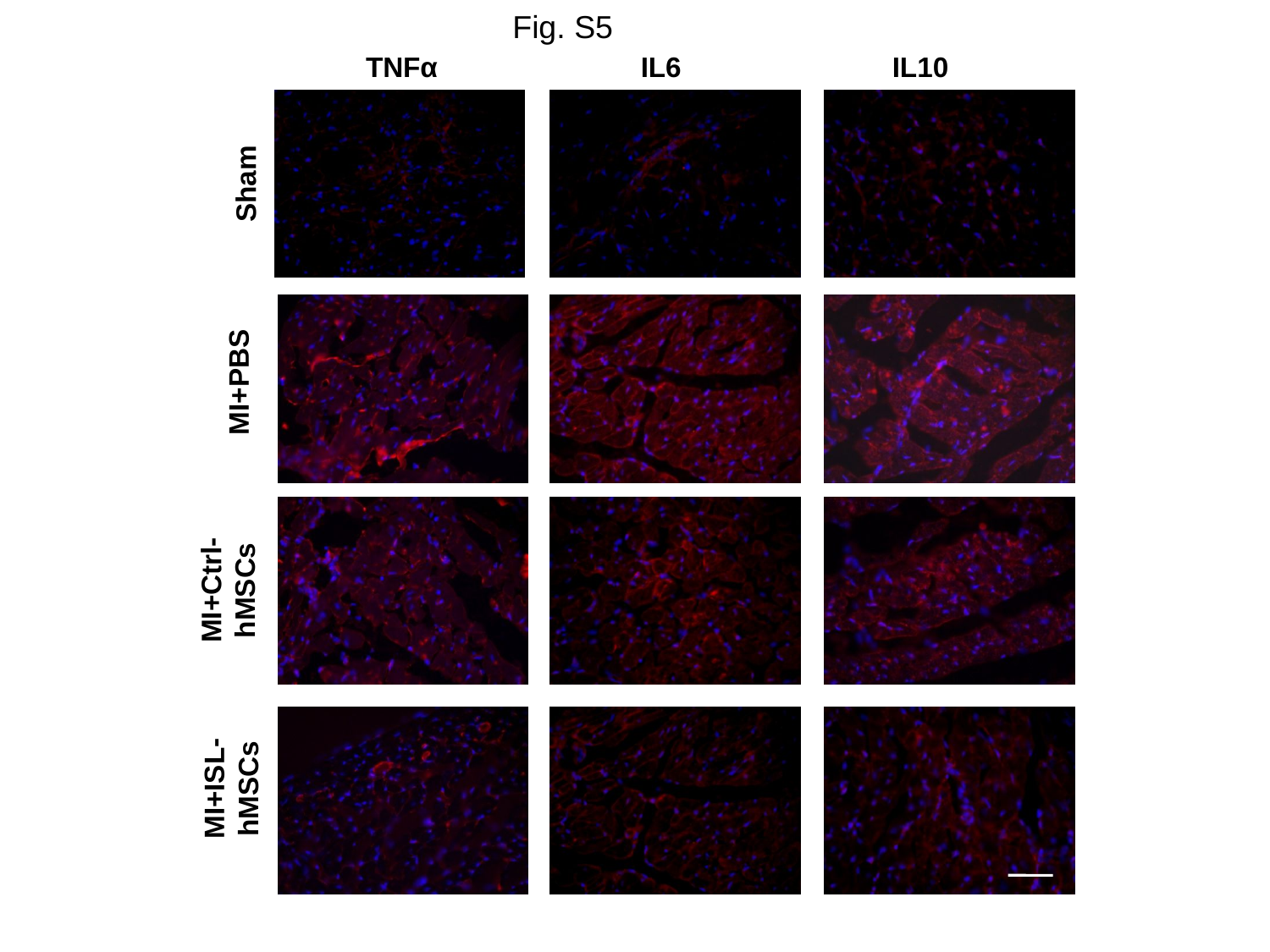

Fig. S5
TNFα IL6 IL10
MI+PBS
MI+Ctrl-hMSCs
MI+ISL-
hMSCs
Sham

## Slide 14
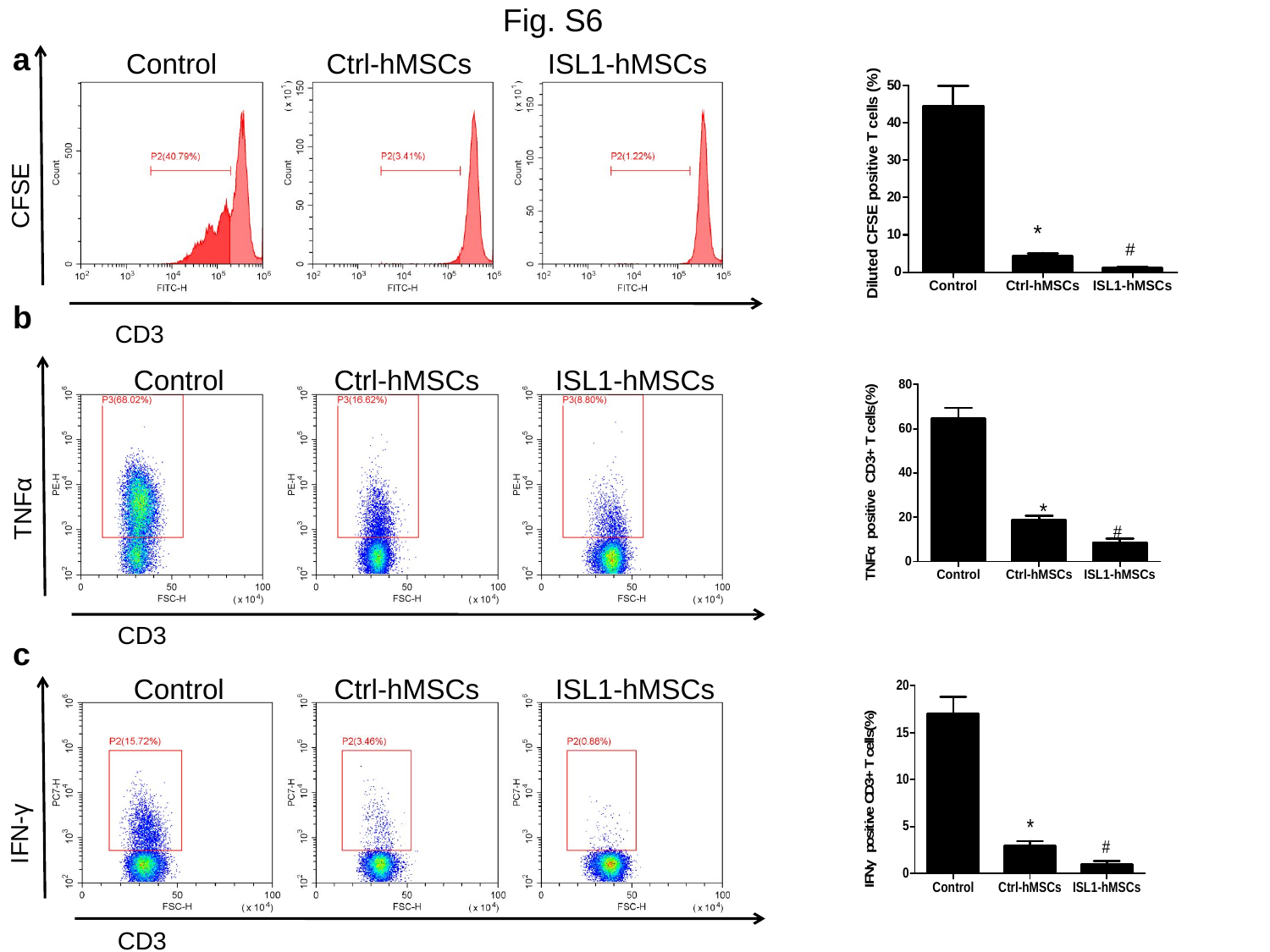

Fig. S6
a
Control
Ctrl-hMSCs
ISL1-hMSCs
CFSE
b
CD3
Control
Ctrl-hMSCs
ISL1-hMSCs
TNFα
CD3
c
Control
Ctrl-hMSCs
ISL1-hMSCs
IFN-γ
CD3

## Slide 15
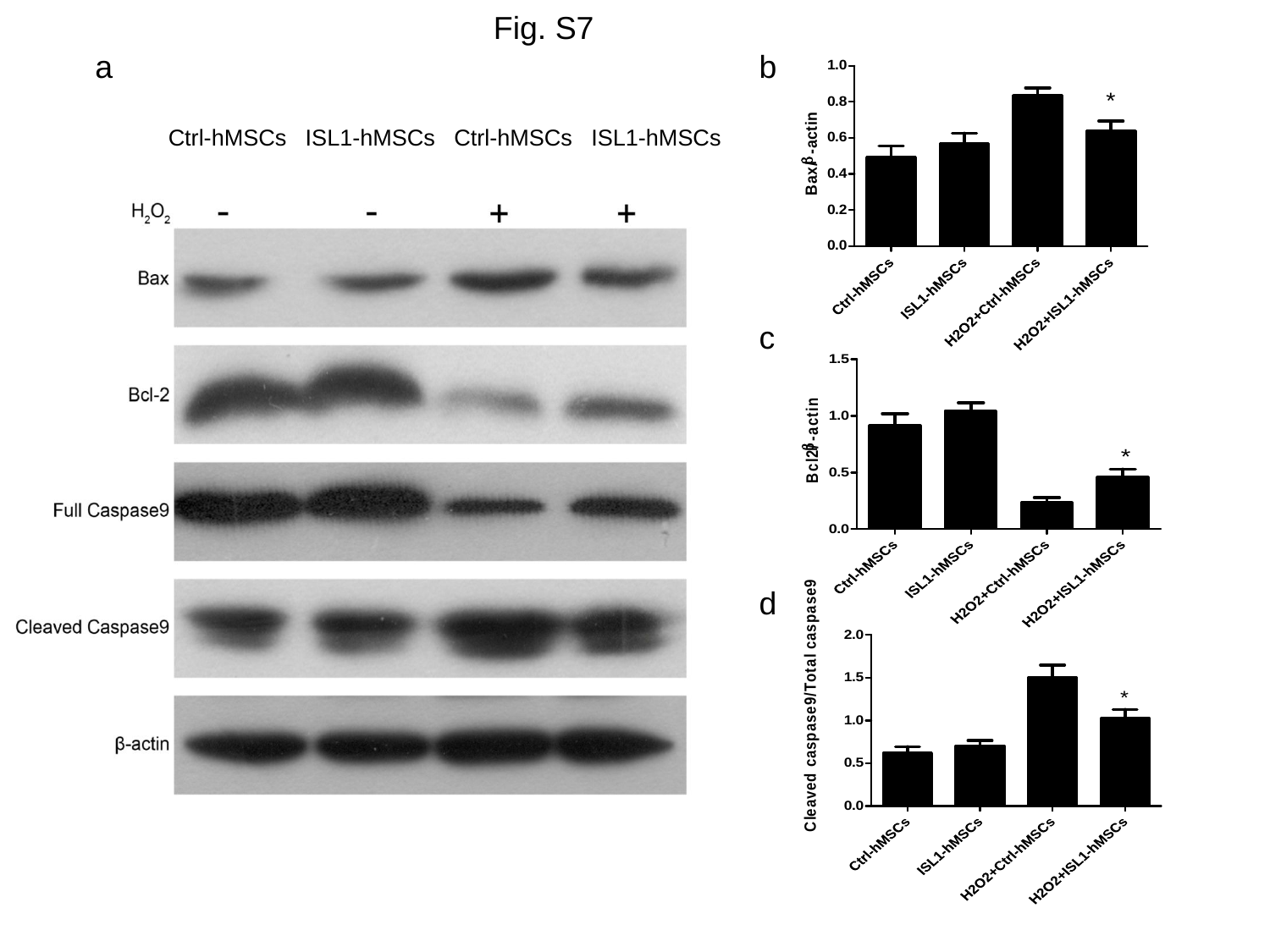

Fig. S7
Ctrl-hMSCs ISL1-hMSCs Ctrl-hMSCs ISL1-hMSCs
a
b
c
d

## Slide 16
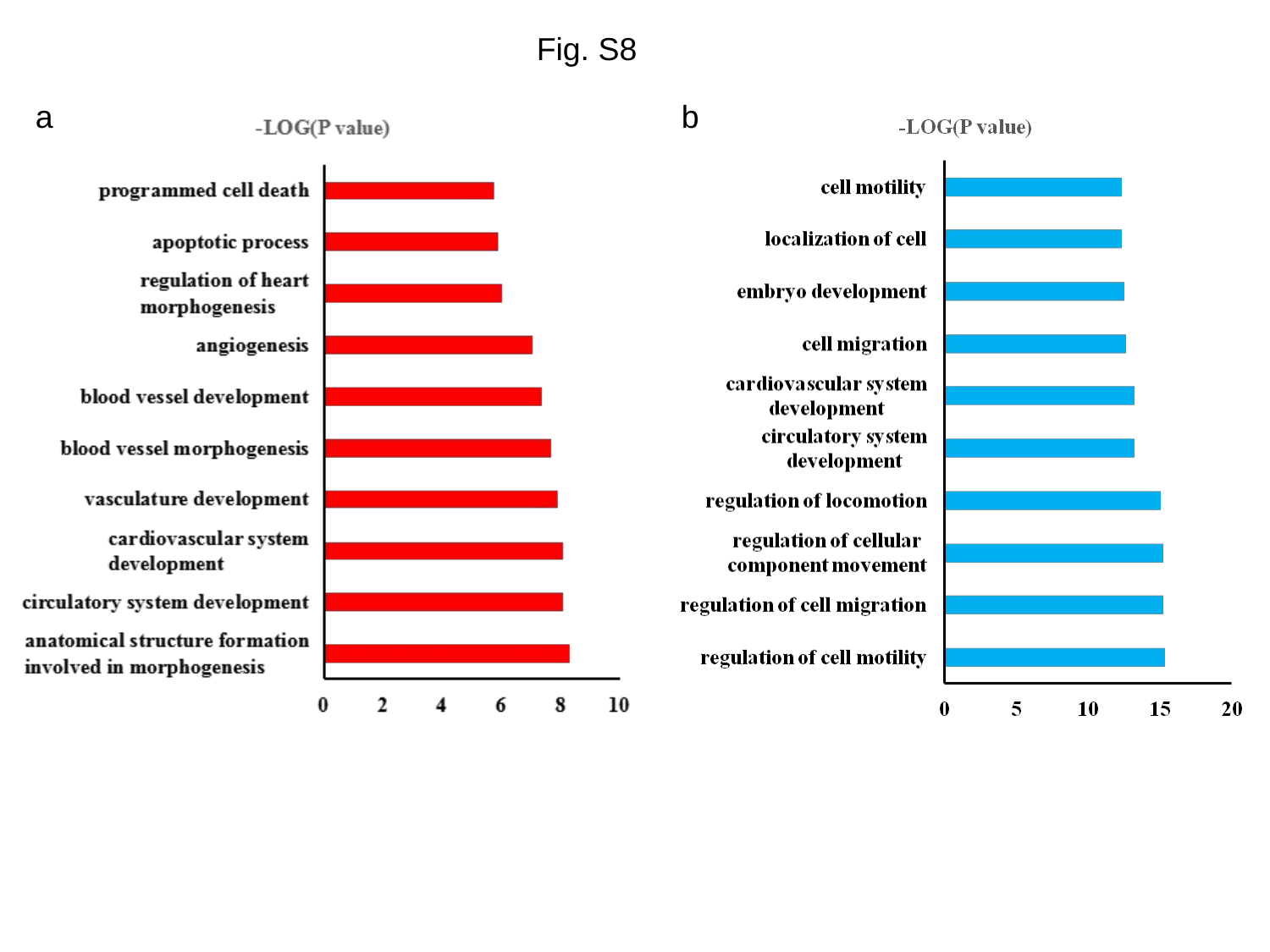

Fig. S8
a
b

## Slide 17
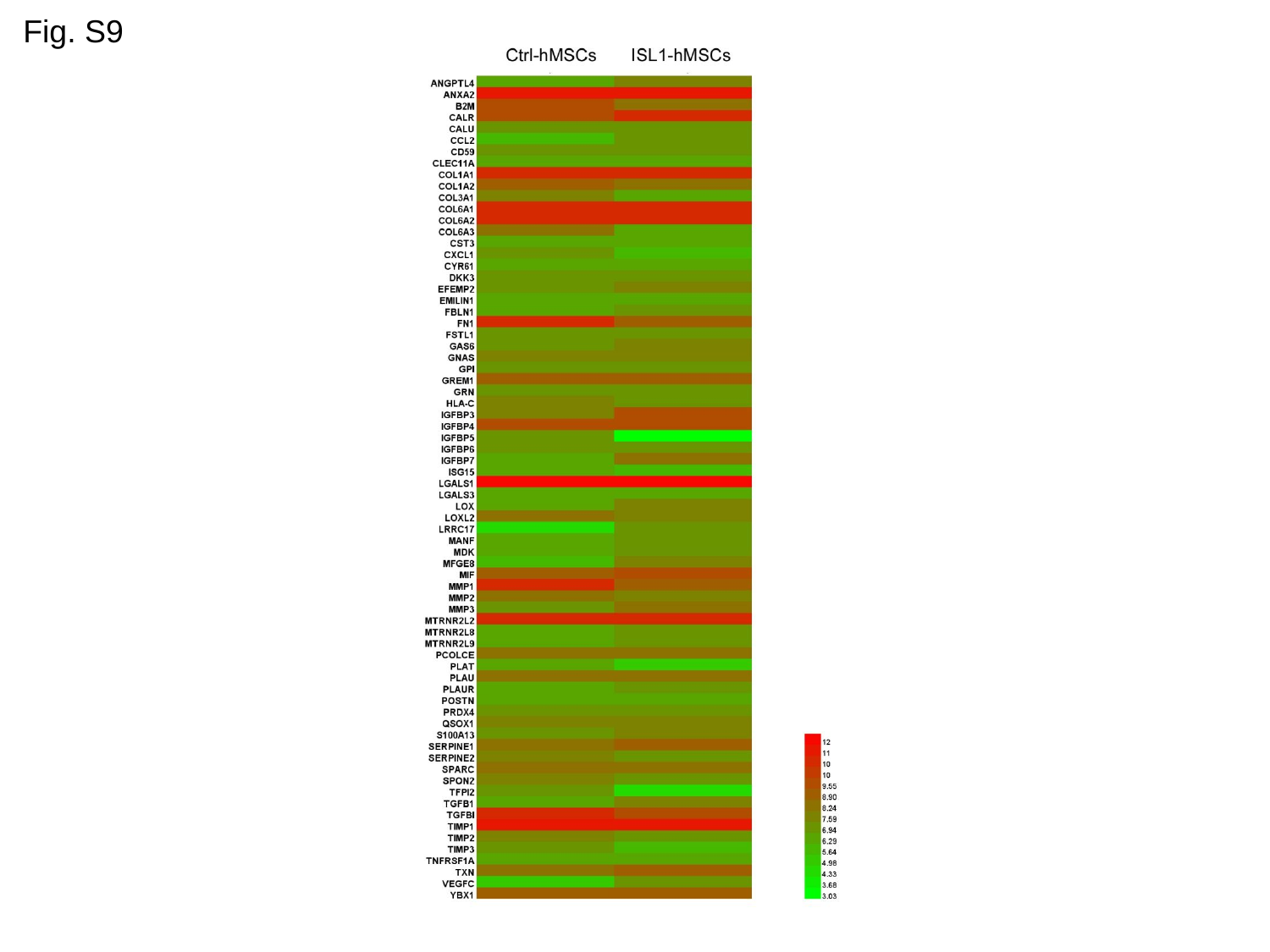

Fig. S9

## Slide 18
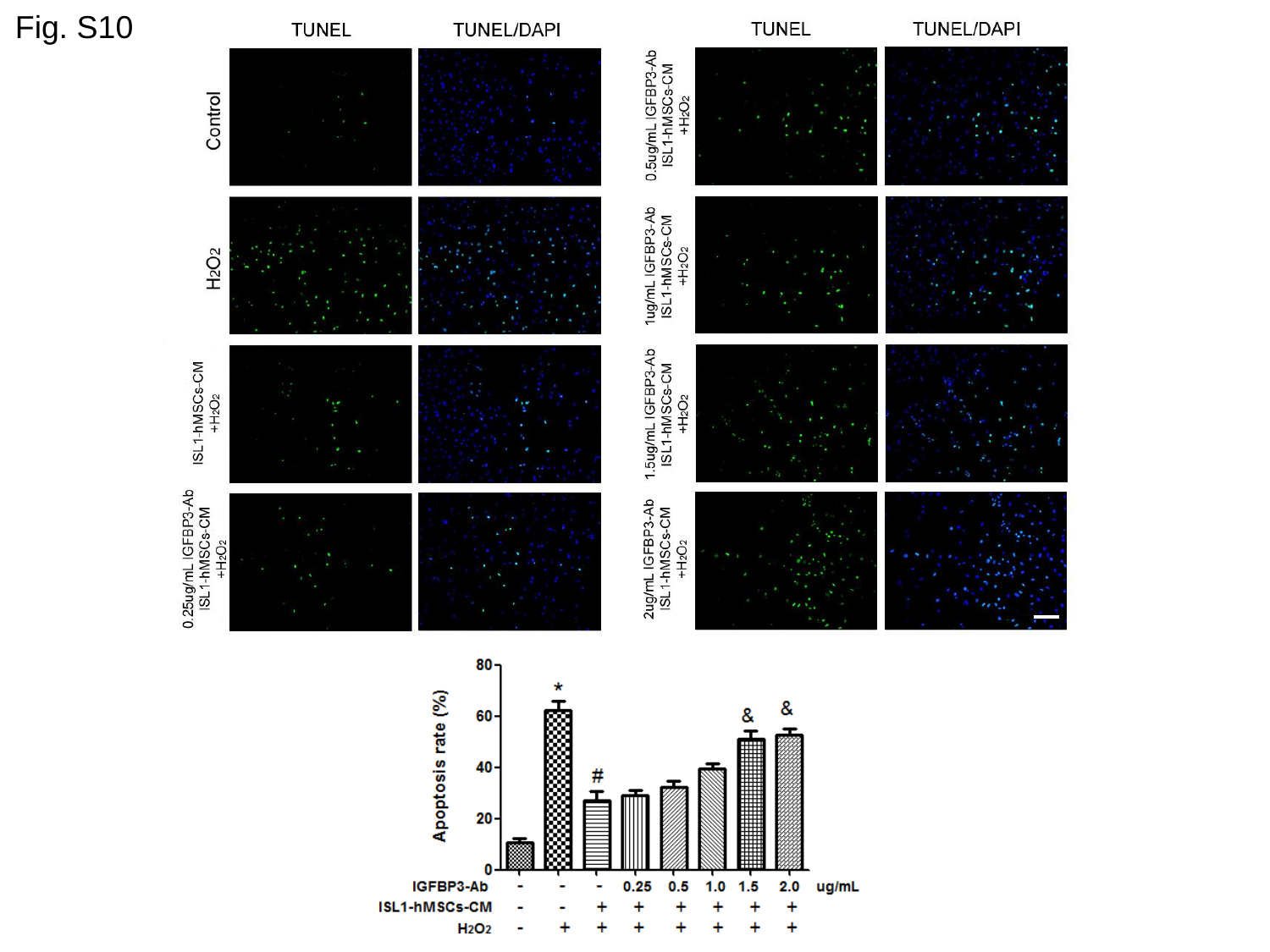

Fig. S10

## Slide 19
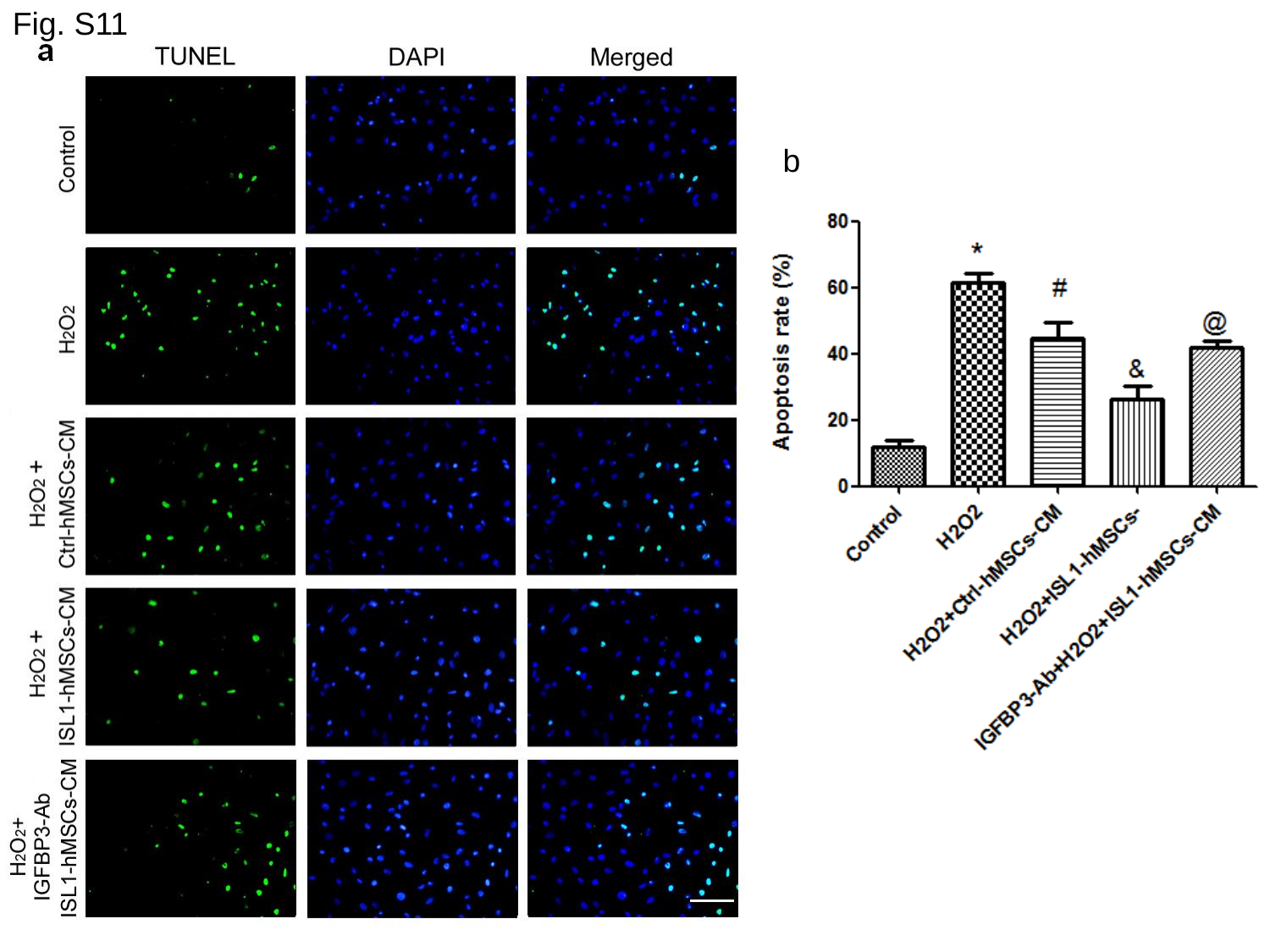

Fig. S11
b
